# A phase I prospective, non-randomized trial of autologous dendritic cell-based cryoimmunotherapy in patients with metastatic castration-resistant prostate cancer

**DOI:** 10.1007/s00262-023-03421-7

**Published:** 2023-03-20

**Authors:** Liv Cecilie Vestrheim Thomsen, Alfred Honoré, Lars Anders Rokne Reisæter, Bjarte Almås, Astrid Børretzen, Svein Inge Helle, Kristina Førde, Einar Klæboe Kristoffersen, Silje Helland Kaada, Guro Kristin Melve, Torjan Magne Haslerud, Martin Biermann, Iris Bigalke, Gunnar Kvalheim, Waqas Azeem, Jan Roger Olsen, Benjamin Gabriel, Stian Knappskog, Ole Johan Halvorsen, Lars Andreas Akslen, Duke Bahn, Klaus Pantel, Sabine Riethdorf, Haakon Ragde, Bjørn Tore Gjertsen, Anne Margrete Øyan, Karl-Henning Kalland, Christian Beisland

**Affiliations:** 1 Centre for Cancer Biomarkers CCBIO, Department of Clinical Science, University of Bergen , Bergen, Norway; 2 Department of Urology, Haukeland University Hospital , Bergen, Norway; 3 Department of Radiology, Haukeland University Hospital, Bergen, Norway; 4 Department of Pathology, Haukeland University Hospital, Bergen, Norway; 5 Department of Oncology, Haukeland University Hospital, Bergen, Norway; 6 Department of Immunology and Transfusion Medicine, Haukeland University Hospital, Bergen, Norway; 7 Department of Cellular Therapy, Oslo University Hospital, Oslo, Norway; 8 Department of Clinical Science, UiB, Bergen, Norway; 9 K.G. Jebsen Center for Genome-Directed Cancer Therapy, Department of Clinical Science, University of Bergen, Bergen, Norway; 10 Centre for Cancer Biomarkers CCBIO, Department of Clinical Medicine, University of Bergen, Bergen, Norway; 11 USC Institute of Urology, Keck School of Medicine, University of Southern California, Los Angeles, CA USA; 12 Institut Für Tumorbiologie, Zentrum Für Experimentelle Medizin, Universitätsklinikum Hamburg-Eppendorf, Hamburg, Germany; 13 Department of Clinical Medicine, University of Bergen, Bergen, Norway

**Keywords:** Metastatic castration-resistant prostate cancer, Immature dendritic cells, Phase I clinical trial, Cryoablation, Immunotherapy, Safety

## Abstract

Metastatic castration-resistant prostate cancer (mCRPC) is an immunologically cold disease with dismal outcomes. Cryoablation destroys cancer tissue, releases tumor-associated antigens and creates a pro-inflammatory microenvironment, while dendritic cells (DCs) activate immune responses through processing of antigens. Immunotherapy combinations could enhance the anti-tumor efficacy. This open-label, single-arm, single-center phase I trial determined the safety and tolerability of combining cryoablation and autologous immature DC, without and with checkpoint inhibitors. Immune responses and clinical outcomes were evaluated. Patients with mCRPC, confirmed metastases and intact prostate gland were included. The first participants underwent prostate cryoablation with intratumoral injection of autologous DCs in a 3 + 3 design. In the second part, patients received cryoablation, the highest acceptable DC dose, and checkpoint inhibition with either ipilimumab or pembrolizumab. Sequentially collected information on adverse events, quality of life, blood values and images were analyzed by standard descriptive statistics. Neither dose-limiting toxicities nor adverse events > grade 3 were observed in the 18 participants. Results indicate antitumor activity through altered T cell receptor repertoires, and 33% durable (> 46 weeks) clinical benefit with median 40.7 months overall survival. Post-treatment pain and fatigue were associated with circulating tumor cell (CTC) presence at inclusion, while CTC responses correlated with clinical outcomes. This trial demonstrates that cryoimmunotherapy in mCRPC is safe and well tolerated, also for the highest DC dose (2.0 × 10^8^) combined with checkpoint inhibitors. Further studies focusing on the biologic indications of antitumor activity and immune system activation could be considered through a phase II trial focusing on treatment responses and immunologic biomarkers.

**Supplementary Information:**

The online version contains supplementary material available at 10.1007/s00262-023-03421-7.

## Introduction

Mortality rates remain high for patients who develop metastatic castration-resistant prostate cancer (mCRPC) [1]. For the last 10–15 years, chemotherapy with a taxane, docetaxel, has been the only life-prolonging option for these patients. Recently, several new treatments have been approved: the anti-androgens, abiraterone and enzalutamide, the taxane, cabazitaxel, the poly (ADP-ribose) polymerase inhibitors, rucaparib and olaparib, the immunotherapy, sipuleucel-T, and the alpha-emitter radium-223 for men with bone metastases. All agents have shown survival benefit for mCRPC in phase III trials, and others are currently under evaluation. Unfortunately, the reported median overall survival (OS) benefit associated with these therapies ranges between 3 and 5 months [2–5].

The immunological checkpoint inhibitor therapies, the cytotoxic T-lymphocyte protein 4 (CTLA-4) inhibitor, ipilimumab and programmed cell death protein 1 (PD-1) inhibitor, pembrolizumab, have demonstrated marked effects on progression-free survival (PFS) in malignant melanoma, renal cell carcinoma, and cancers with DNA repair deficiencies [6, 7]. mCRPC is considered an immunologically cold cancer [8]. Notwithstanding, while previous trials on ipilimumab and pembrolizumab treatment in mCRPC patients failed to demonstrate clinical benefit [9–11], two recent immune checkpoint inhibitor therapy trials indicate clinical effects including increased OS among a defined patient subset [12, 13].

An essential part of the immune response is mediated by dendritic cells (DCs), which are antigen-presenting cells that in their immature state recognize and process tumor-associated antigens. Early-phase trials of DC-based cancer immunotherapies have demonstrated the treatment as safe and feasible with anti-tumor immune activation, but with limited clinical responses [14, 15]. Currently, several DC-based vaccine trials in prostate cancer are ongoing, including for the US Food and Drug Administration-approved sipuleucel-T [4].

The capacity of malignancies to inactivate DCs and effector T cells and evade the circulating antitumor immune responses is challenging the development of immune-modulating therapies. The T cell subset, regulatory T-lymphocyte (Treg), plays a dual role of mediating immune tolerance and restricting the antitumor immunity [16]. In cancer treatment, medical interventions can induce a Treg expansion, which decreases the cytotoxic effects. Subclinical doses of the chemotherapeutic drug, cyclophosphamide, deplete the Treg fraction of T cells in tumors and appear to restore the anti-tumor effects of the adaptive immune system [17].

The heterogeneous expression of molecular targets on cells within and between tumors induces different treatment sensitivities and accordingly poses a challenge for targeted therapies. Previously, in vitro matured DCs have been trained to destroy mainly tumor cells expressing preselected antigens, but additionally generate bystander effects, where untreated cells are biologically altered by stress signals from directly treated cells to mirror these by exhibiting similar effects like reduced cell survival and genomic instability [18]. Combination therapies show promise to counteract immune evasion and partly overcome the tumor heterogeneity, for instance, in renal cell carcinoma and malignant melanoma [6, 7].

Cancer cell heterogeneity is one of the most fundamental problems of cancer therapy. By combining cryoablation with intratumoral DC injection, cryoimmunotherapy (CryoIT) exposes the entire individualized collection of tumor-associated antigens to immature DCs (iDCs), which consequently are positioned to tackle the cellular heterogeneity. Cryoablation is a process where tissue destruction is initiated by freezing solid tumors to -40 °C in one or several freeze–thaw cycles. Cryoablation has been investigated in localized, locally recurrent, and metastatic prostate cancer [19, 20]. The destruction of tumoral tissues by freezing causes direct cell damage, necrosis and apoptosis. Consequently, two important effects are induced: the release of tumor-associated antigens that are engulfed by iDCs, and the generation of local inflammation with accumulation of proinflammatory cytokines. CryoIT positions iDCs in the proinflammatory microenvironment where any tumor-associated antigen can be processed by maturing DCs, which subsequently migrate to the draining lymph nodes to stage a systemic attack on cancer cells. While some evidence of potential therapeutic effects of combining cryoablation with iDC treatment for mCRPC exists [21], it has not previously been evaluated in the setting of a clinical trial.

The primary aim of this phase I clinical trial of pre-treated mCRPC was to examine the safety and tolerability of the novel combination of cancer tissue cryoablation and intratumoral injection of autologous iDCs with and without immunological checkpoint inhibitor enhancement. Secondary and explorative endpoints included time to progression, survival, and monitoring of immune responses and circulating tumor cells.

## Materials and methods

### Participants

Eligible patients had mCRPC with metastases and an Eastern Cooperative Oncology Group (ECOG) performance status of 0–1, adequate organ function, no known hypersensitivity to vaccines or components of the cell therapy, and no contraindications to surgery (Supplementary S1B). The exclusion criteria contained immunodeficiency, other active malignancy, and recent or ongoing anti-tumor treatment. Prior radiotherapy and treatment with androgen deprivation and androgen receptor targeting drugs were accepted (Supplementary S1C).

### Study design and data capture

**Fig. 1:**
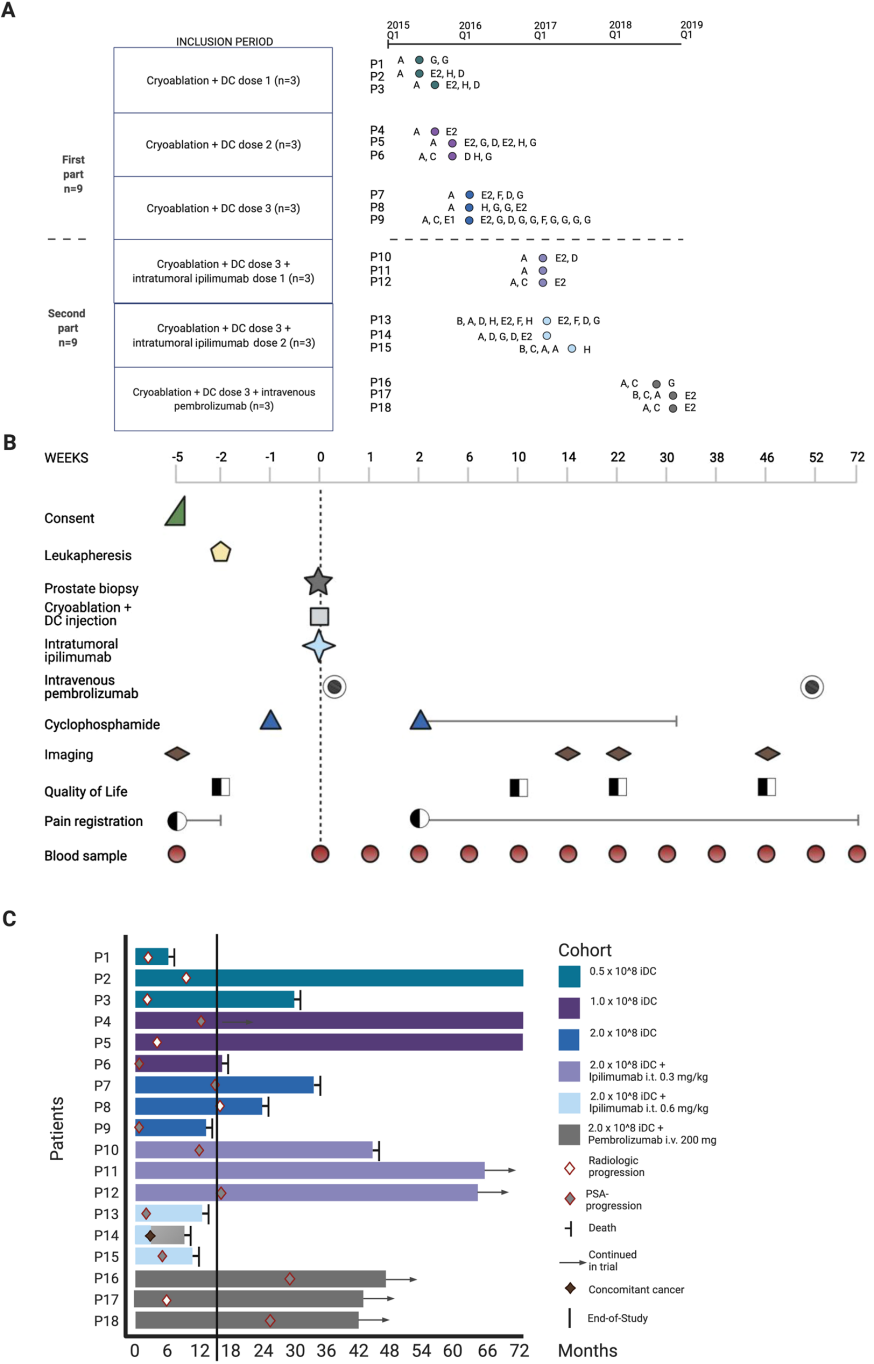
Study overview. **A** Graphical representation of the inclusion period of participants and cancer-directed treatment lines. The two cohort parts are separated by the horizontal dashed line. Time of inclusion according to year, quartile, and dose of study drugs is indicated by colored dots. Cancer-directed treatment received by each participant prior to inclusion (to the left of dots) and after disease progression during the trial participation period (to the right of dots) is listed as letters. *A*; GnRH agonist (+ initial 4 weeks with bicalutamide), *B*; GNRH antagonist, *C*; early chemotherapy (≤ 3 months after diagnosis), *D*; late chemotherapy (> 3 months after diagnosis), *E*; antiandrogen monotherapy (E1; bicalutamide, E2; enzalutamide), *F*; androgen-signaling inhibitor (abiraterone), *G*; external beam radiation (EBRT) for symptomatic disease, H; EBRT combined with i.v. radium-223**. B** Clinical trial design. Procedures performed as part of the CryoIT trial are listed to the left, and the symbols indicate at which time points during the trial the participants had each procedure done. The vertical dashed line at 0 weeks indicates the time of cryoablation and autologous dendritic cell injection. **C** Swimmer plot with response patterns. Each bar shows the response of one patient. The 0 on the horizontal axis indicates time of CryoIT treatment. The follow-up period in months is given along the horizontal axis. The vertical line indicates End-of-Trial 72 weeks after CryoIT. Of the 18 patients, 17 had subsequent progressive disease, and ten died. One participant (P14) had stable prostate cancer but developed concomitant malignant melanoma with rapid progression leading to death. This patient was only included in the analyses of the safety of the treatment and analyses where results were annotated by participant ID. Among the patients who were still in follow-up at the time of data cut-off, eight had progressed while one still had clinical treatment benefit according to the last follow-up of 48 months after treatment. Figure created with BioRender.com, i.t. intratumoral injection, i.v. intravenous infusion

This phase I open-label interventional study recruited patients at Haukeland University Hospital, Bergen, Norway. The study consisted of two study parts, investigating the safety and tolerability of combining either cryoablation and iDC treatment (first part), or cryoablation, iDC treatment and immune checkpoint inhibitor therapy (second part), illustrated in Fig. 1 and the CONSORT Flow diagram (Supplementary Appendix A). Each part was to include minimum nine mCRPC patients with an intact prostate gland and radiologically confirmed metastases. In the first part, the dose of intratumorally distributed iDCs was increased according to the traditional 3 + 3 design for Phase I cancer trials to a predefined maximum dose of 2.0 × 10^8^ iDCs [22]. In the second part, participants were to receive either ipilimumab (Supplementary S4) intratumorally in a 3 + 3 design, increasing from 0.3 to 0.6 mg/kg if the adverse event (AE) profiles allowed, or two intravenous injections of 200 mg pembrolizumab (Supplementary S4).

Generally, participants attended 14 study-specific visits from inclusion to 52 weeks after the CryoIT, with a follow-up period of 72 months. The CryoIT procedure was performed during visit 4 (day 0). Medications, vital signs, and results from the physical examinations and routine laboratory blood analyses were protocolled. Safety assessments were performed during all post-treatment visits. Imaging was performed concomitantly by MRI of the spinal column, pelvic region and prostate gland, whole-body PET/CT with F18-fluorodeoxyglucose, and radionuclide bone scan with [^99m^Tc]methyldiphosphonate. Radiologic examinations were performed at inclusion, and 14, 22, and 46 weeks after treatment (Fig. 1, Supplementary Fig. S2).

Moreover, health-related quality of life (HRQoL) forms, visual analog scale (VAS) pain measurements, and blood samples were collected as depicted in Fig. 1B. The study data were captured in the software WebCRF3 (v.2016–05-23) [23].

### Procedures

Leukapheresis was performed 14 days prior to CryoIT followed by monocyte enrichment using the ELUTRATM System (Supplementary S2). Autologous iDCs were produced according to protocol (Supplementary S2).

Cryoablation of the prostate was performed under general anesthesia and ultrasound guidance as a cyclic freeze–thaw process (Supplementary S3). Directly prior to the first freezing cycle, eight core prostate biopsies were sampled transrectally. Succeeding cryoablation, the pre-specified number of viable iDCs was injected intratumorally into the prostate. All patients received cyclophosphamide, 300 mg/m2 intravenously three days prior to CryoIT, and low oral doses (50–100 mg/day) metronomically every other week from week 2 to week 26 post-treatment to avoid Treg increases that might diminish incipient immune responses (Supplementary S5, Fig. 1).

### Assessment of safety and toxicity

AEs were recorded, and their severity graded according to the Common Terminology Criteria for Adverse Events v3.0. Clinical investigators evaluated the severity and relatedness of any registered AEs to the trial participation, study drug(s) and ablative procedure. Any AEs that could be related to trial participation were defined as treatment emergent. Any severe AEs, defined as AEs ≥ grade 3, were reported to the sponsor within 48 h. To establish the maximum tolerated dose and recommended phase II trial dose of iDCs, the toxicity was defined as dose limiting if patients experienced any persistent grade 4 toxicity. Maximum tolerated dose was defined as the dose level below which dose limiting toxicities were seen in ≥ 1 of 3 subjects. Given the observed tolerability of DC-based therapy in part 1, subjects in part 2 were treated with the highest iDC dose. Dose-limiting toxicity of cryoablation plus iDC treatment was evaluated separately (*n* = 9) and combined with intratumoral ipilimumab (Bristol-Myers Squibb, ATC-no. L01XC11) (*n* = 6) or systemic pembrolizumab (Merck Sharp&Dohme B.V., ATC-no. L01XC18) (*n *= 3).

### Assessment of response

Radiologic responses were assessed by internal expert review. MRI and PET/CT images were evaluated according to the RECISTv1.1. criteria [24]. Bone scintigraphies were evaluated according to the Prostate Cancer Working Group (PCWG)2 criteria [25]. Patients were defined as having clinical treatment benefit 22 and 46 weeks after CryoIT if they demonstrated complete or partial responses or sustained stable disease. A strict definition of clinical benefit was selected, only annotated to patients when all three imaging modalities demonstrated at least stable disease.

Prostate-specific antigen (PSA) levels should change at least 25% and display an absolute increase/decrease of 2 ng/mL to define PSA-related progression or improved disease status [20]. PFS and OS were defined from the date of CryoIT until either radiological and/or PSA-based progression, death, or the date of last follow-up.

Lactate dehydrogenase (LDH) and alkaline phosphatase (ALP), blood-based markers of prostate cancer tumor load and skeletal involvement, were measured sequentially at the routine laboratory.

Circulating tumor cells (CTCs) were enumerated pre-treatment and during all follow-up visits using the CellSearch® System (Menarini Silicon Biosystems) according to the producer's standardized procedure (Supplementary S8). CTC responses were grouped as CTCs = 0 or CTCs > 0 independent of pre-treatment values. The response was estimated for two time points: at first available CTCs measurements after the CryoIT, and two weeks after treatment.

Patient-reported outcomes were collected using the European Organization for Research and Treatment of Cancer (EORTC) QLQ-C30 questionnaire [26]. HRQoL was analyzed according to the EORTC scoring manual (Supplementary S6). Associations between HRQoL and CTC presence were investigated with CTC as a dichotomous variable (absence/presence).

Pain was evaluated by VAS pain logs prior to treatment, and from week two after CryoIT (Supplementary S6).

### Translational outcomes

Flow cytometry analyses of immune cells were conducted according to local routine (Supplementary S7) utilizing BD FACSCanto II (3 lasers) and BD FACSDiva software v.8.0.1 (BD Biosciences).

Dedicated uropathologists re-examined the formalin-fixed paraffin-embedded (FFPE) primary diagnostic biopsies, assigning all samples a histologic subtype and International Society of Urological Pathology (ISUP) grade group. Furthermore, FFPE hematoxylin- and eosin-stained slides from all eight study biopsies, collected prior to CryoIT from each participant, were examined and graded (Supplementary S8).

For each participant, the study biopsy with the most tumor tissue and the highest Gleason pattern was selected for immunohistochemistry analyses for T cells. The corresponding fresh frozen biopsy was selected for the two genetic panels and the T-cell receptor (TCR) sequencing (Supplementary S8 and S10).

The biopsied tissue was stained for the four MMR proteins: MSH2, MSH6, PMS2 and MLH1, using the platform Ventana BenchMark Ultra (Roche, Basel, Switzerland) and detection system OptiView (Supplementary S9). MSI was diagnosed when minimum one of four MMR proteins was unstained in tumor nuclei, while positive staining < 10% classified as equivocal.

DNA was isolated from each sample using the Qiagen AllPrep DNA/RNA Mini Kit (Qiagen, Hilden, Germany). Targeted parallel sequencing for library preparation (Agilent SureSelect XT-kit, Agilent) was performed on DNA from tumor tissue and matched peripheral blood. For the 360 gene panel targeted enrichment was performed using RNA baits (SureSelect, Agilent) against the coding regions of 360 cancer-related genes [27]. Libraries were sequenced on a MiSeq instrument (Illumina, San Diego, California, USA) aiming at average depths of 200x. Potential MSI was assessed by the Promega MSI analysis system (v.1.2, Promega, WI, USA) according to the manufacturer’s instructions. The number of altered mononucleotide markers classified the tumors as MSI-high (2/5), MSI-low (1/5), or microsatellite stable (MSS) (0/5).

TCR clonotypes were enumerated according to sequence counts. The 200 clonotypes with the highest sequence counts two and six weeks after CryoIT were compared separately with results from pre-treatment samples. Novel clonotypes were those that were undetectable prior to, but detectable after the CryoIT. For pre-treatment samples, the maximum clonal frequencies were used. Expanded clonotypes were pre-treatment clonotypes that expanded > fivefold after treatment. The sum of the frequencies of all identified TCR clonotypes in the entire data set defined the clonal space, while the clonal space percentage of a clonotype was calculated as the percentage of the total number of identified clonotypes.

### Data processing and bioinformatics analysis

For the 360 gene panel analyses, raw sequence data were aligned to the human reference genome (Build-UCSC hg19) using BWA. Somatic single nucleotide variants were detected by application of CaVEMan and insertions/deletions were detected using Pindel [28]. All somatic mutations were validated by manual inspection in Integrative Genomics Viewer. Allele-specific copy number analysis and estimation of purity and ploidy were performed using FACETS [29].

The tumor mutational burden (TMB) analyses were performed by the TSO500 assay (NextSeq platform, Illumina) at the Science for Life Laboratory, Uppsala University, Sweden, (Supplementary S9) according to the producer´s protocol.

Bioinformatic processing of the TCR sequencing results was performed by HS Diagnomics (Berlin, Germany). The libraries were based on a 2-step PCR system using gene-specific primers for *TRBV* and *TRBJ*. The final TCR sequencing libraries were pooled and sequenced on Illumina MiSeq instruments (Illumina) using 2 × 150 paired-end reads and 20% PhiX spike. On average, 700,000 reads per library was targeted.

### Statistical considerations

In this phase I clinical trial, sample sizes were not based on statistical methods. The aim of both trial parts was to evaluate the toxicity profile rather than formally to demonstrate any efficacy endpoints. The database cut-off date was April 30, 2021, when all participants had reached 24 months of follow-up, and OS data were updated last October 5, 2022.

Descriptive statistics were used to categorize AEs according to their nature and severity. Furthermore, descriptive statistics were applied to demographic data, CTC results, and generation of the global HRQoL spider plot and line graphs, to demonstrate changes in TCR clonotype counts and longevity, and to depict percentage changes by waterfall plots.

Unless otherwise stated, all statistical testing between groups was made by Fisher’s exact tests, two-sided Mann–Whitney U tests, or Kruskal–Wallis tests.

Statistical significance was defined as *p* < 0.05. Due to the small sample size and hypothesis-generating nature of the analyses, results should be interpreted with caution. Adjustments for multiple testing were not performed.

PFS and OS were estimated at database cut-off with corresponding 95% confidence intervals estimated by the Kaplan–Meier method and independent groups compared by log-rank tests. Data analyses were performed either in R v.3.6.0 or higher [30], Excel v.16.21 (Microsoft), or IBM SPSS Statistics v.26 (IBM Corp.).

## Results

### Patients and treatment

Between May 2015 and November 2018, 21 patients were consented and screened. Eighteen were admitted to the trial, as three patients exhibited one exclusion criterion. The median follow-up was 38.3 months (interquartile ranges (IQR) 14.1–60.9). Baseline characteristics of participants are summarized in Table 1.

**Table 1:** Baseline characteristics

	Patients (*n* = 18)
Age, years	70 (62–74)
*ECOG performance status*	
0	16 (89%)
1	2 (11%)
Time from primary diagnosis, months	30.5 (14.1–49.7)
*Metastasis site at inclusion*	
Bone	17 (94%)
Lymph nodes	1 (6%)
Other	0 (0%)
Body mass index, kg/m^2^	27 (26–30)
Prostate-specific antigen, ng/mL	8.4 (4.7–39.4)
Alkaline phosphatase, U/L	77 (66–100)
Lactate dehydrogenase, U/L	189 (174–195)
*Number of previous therapeutic regiments, n (%)*	
1	9 (50%)
2	4 (22%)
≥ 3	5 (28%)
Prior antiandrogen + GnRH agonist or GnRH antagonist therapy	18 (100%)
GnRH agonist (+ initial 4w bicalutamide)	15
GnRH antagonist	3
Prior platinum-based chemotherapy for metastatic disease	9 (50%)

Results are given as medians with 1st-3rd interquartile ranges in parentheses, or numbers with percentages in parentheses

*ECOG* = Eastern Cooperative Oncology Group performance status; *U/L* = units per liter; *GnRH* = gonadotropin-releasing hormone

At diagnosis, the participants demonstrated median Gleason score 8, perineural invasion (100%), and vascular invasion (33%) (Supplementary Table S2). All patients had intact prostate glands, and were previously treated by at least one gonadotropin-releasing hormone (GnRH) analogue (GnRH agonists; *n* = 15, GnRH antagonists; *n* = 3). Additional prior therapy lines are documented in Fig. 1A and Supplementary Fig. S1.

Leukapheresis was performed 24 (IQR 20–30) days prior to CryoIT. The first three patients received 5 × 10^7^ autologous iDCs, the next three 1.0 × 10^8^ iDCs, and the remaining participants 2.0 × 10^8^ iDCs. Patients 10–12 and 13–15 additionally received a single dose of ipilimumab 0.3 mg/kg and 0.6 mg/kg, respectively, injected into the cryoablated prostate tumor following the administration of iDCs. Subjects enrolled as numbers 16–18 received 200 mg pembrolizumab intravenously on either day 1 (*n* = 2) or day 77 (*n* = 1) after CryoIT, with a repeat dose after 320/320/356 days (Fig. 1A, Supplementary S2B and S4). iDC viability ranged from 75–99% (median 89.5, IQR 85.3–93.0). The first nine and the last nine patients included in the trial differed at baseline in median body mass index (*p* = 0.02), and levels of LDH (*p* = 0.02), hemoglobin (*p* = 0.04) and leukocytes (*p* = 0.02). Otherwise, all demographic and biomarker measurements were comparable between patient groups (Supplementary Table S1).

### Safety

In total, 73 AEs were reported, whereof 32 were possibly or probably associated with participation in the trial. No dose-limiting toxic effects were recorded. The most frequently reported events were mild or moderate urinary tract reactions, administration site reactions, nausea, and influenza-like and common cold symptoms. Only two grade 3 AEs associated with the treatment were reported: one urine retention requiring hospitalization and one pelvic osteomyelitis requiring protracted intravenous antibiotics before full recovery (Table 2). No apparent correlation was observed between occurrences of AEs, iDC dose or the addition or dose of checkpoint inhibitors. Therefore, all subjects in the expansion cohort received the highest dose of 2.0 × 10^8^ iDCs.

**Table 2:** Treatment-emergent adverse events

	Total cohort (*n* = 18)		Cryoablation + iDC dose 1 (*n* = 3)		Cryoablation + iDC dose 2 (*n* = 3)		Cryoablation + iDC dose 3 (*n* = 3)		Cryoablation + iDC + CTLA-4i dose 1 (*n* = 3)		Cryoablation + iDC + CTLA-4i dose 2 (*n* = 3)		Cryoablation + iDC + PD-1i (*n* = 3)	
	Grade 1–2	Grade 3	Grade 1–2	Grade 3	Grade 1–2	Grade 3	Grade 1–2	Grade 3	Grade 1–2	Grade 3	Grade 1–2	Grade 3	Grade 1–2	Grade 3
Any treatment-emergent adverse event	30	2	4	0	5	1	5	1	7	0	2	0	7	0
Urinary retention	6	1			1	1	2		1		1		1	
Lower urinary tract infection	1	0					1							
Urinary incontinence	2	0			1								1	
Pollakiuria	2	0									1		1	
Anemia	1	0					1							
Nausea	2	0	1		1									
Fecal incontinence	2	0	1										1	
Constipation	2	0					1		1					
Suprapubic pain	1	0			1									
Rectal pain	1	0	1											
Hemorrhoids	1	0							1					
Bladder spasm	1	0											1	
Influenza-like illness	2	0			1				1					
Common cold	2	0			1				1					
Sore throat	1	0							1					
Dry mouth	1	0											1	
Vitamin B12 deficiency	1	0			1									
Osteomyelitis	0	1						1						
Increased body rigidity	1	0											1	
Angina pectoris	1	0	1											
Facial rash	1	0							1					

Data are numbers in all treated patients. Treatment-emergent adverse events definitely, probably, or possibly attributed to cryoablation and/or iDC and/or PD-1i/CTLA-4i are shown. No grade 4 or grade 5 adverse events were attributed to treatment as definite, probable, or possible. iDC = immature dendritic cells. CTLA-4i = cytotoxic T-lymphocyte-associated protein 4 inhibitor. PD-1i = programmed cell death protein 1 inhibitor

### Response to therapy

At inclusion, 17/18 participants had skeletal lesions visible on scintigraphy, while 16/18 patients had evaluable visceral disease in the form of prostatic tissue tumors. Several patients had PET-positive lymph nodes. Long-term (> 46 weeks) clinical benefit were achieved by 6/18 (33%) participants. Out of 12, 1 patient who progressed was not followed radiologically due to rapid disease progression and malaise (Fig. 1C), while one patient was diagnosed with concomitant metastatic malignant melanoma, leading to early death. This patient (P14) was only included in analyses of treatment safety, and per individual CTC enumeration and TCR sequencing data (Table 2, Fig. 3). At the time of analyses, the median PFS was 10.5 months (95% CI 0–23) and OS was 40.7 months (95% CI 13.5-NA) (Fig. 1).

**Fig. 2:**
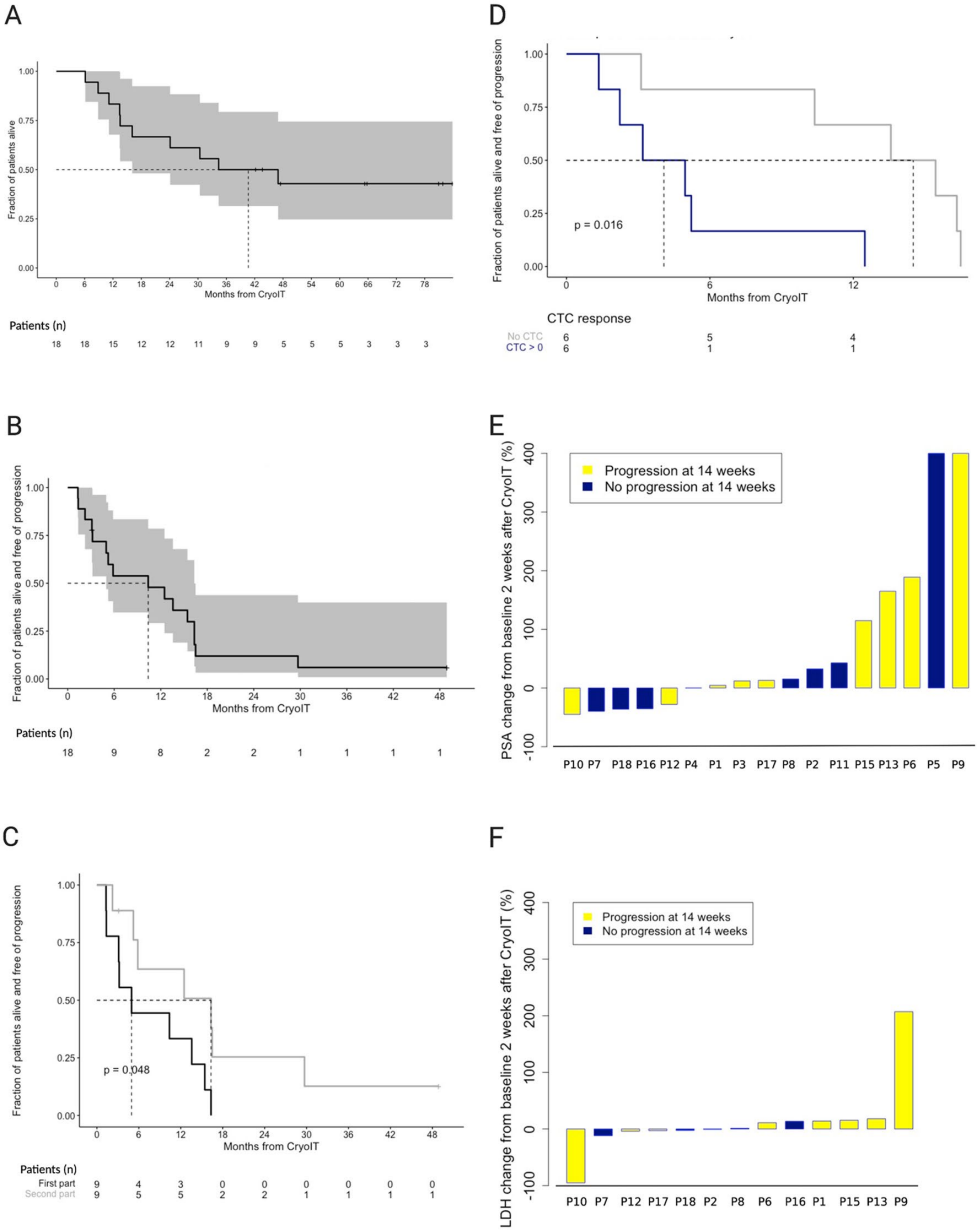
Clinical, laboratory and radiological outcomes. **A–D** Survival estimates visualized by Kaplan–Meier curves. Progression was estimated based on radiologic images and PSA changes. The patient with concomitant cancer development (P14) was excluded from the analyses. **A** Overall survival in the total cohort (*n* = 18). **B** Progression-free survival (PFS) in the total cohort (*n* = 18). **C** PFS in the two parts of the trial, the first nine participants in black (First part), and the participants included as number 10–18 in gray (Second part). **D** CTC response two weeks after the CryoIT procedure. Grouped as No CTC (CTC = 0) or CTC > 0 independent on pre-treatment values. The p-values resulting from the comparisons in **(C)** and **(D)** are listed in the plots. For all four plots, the months of survival after the CryoIT are given along the x-axis. Fractions of the total patient cohort are listed along the y-axis. The dotted lines indicate the time point when 50 percent of the cohort had reached the end point. The number of patients used for the analyses are shown below the x-axis. The 95% confidence intervals are illustrated by a gray area in **A** and **B**. **E–F** show waterfall plots illustrating changes from baseline. Changes in PSA **E** and lactate dehydrogenase (LDH) **F** two weeks after CryoIT are shown. The bars indicating patients with progression are blue, and those with non-progressive disease at week 14 are yellow. Progression was defined radiologically and/or based on PSA increases > 25% and an absolute increase of 2 ng/mL

When changes were measured two and six weeks after CryoIT, differences were observed between participants who had progressed radiologically and those with clinical benefit 22 weeks after treatment (PSA (*p* = 0.003 and *p* = 0.002 after two and six weeks, respectively), LDH (*p* = 0.01 after six weeks)) (Fig. 2D-E, Supplementary S12, Supplementary Table S9, Supplementary Fig. S10).

**Fig. 3:**
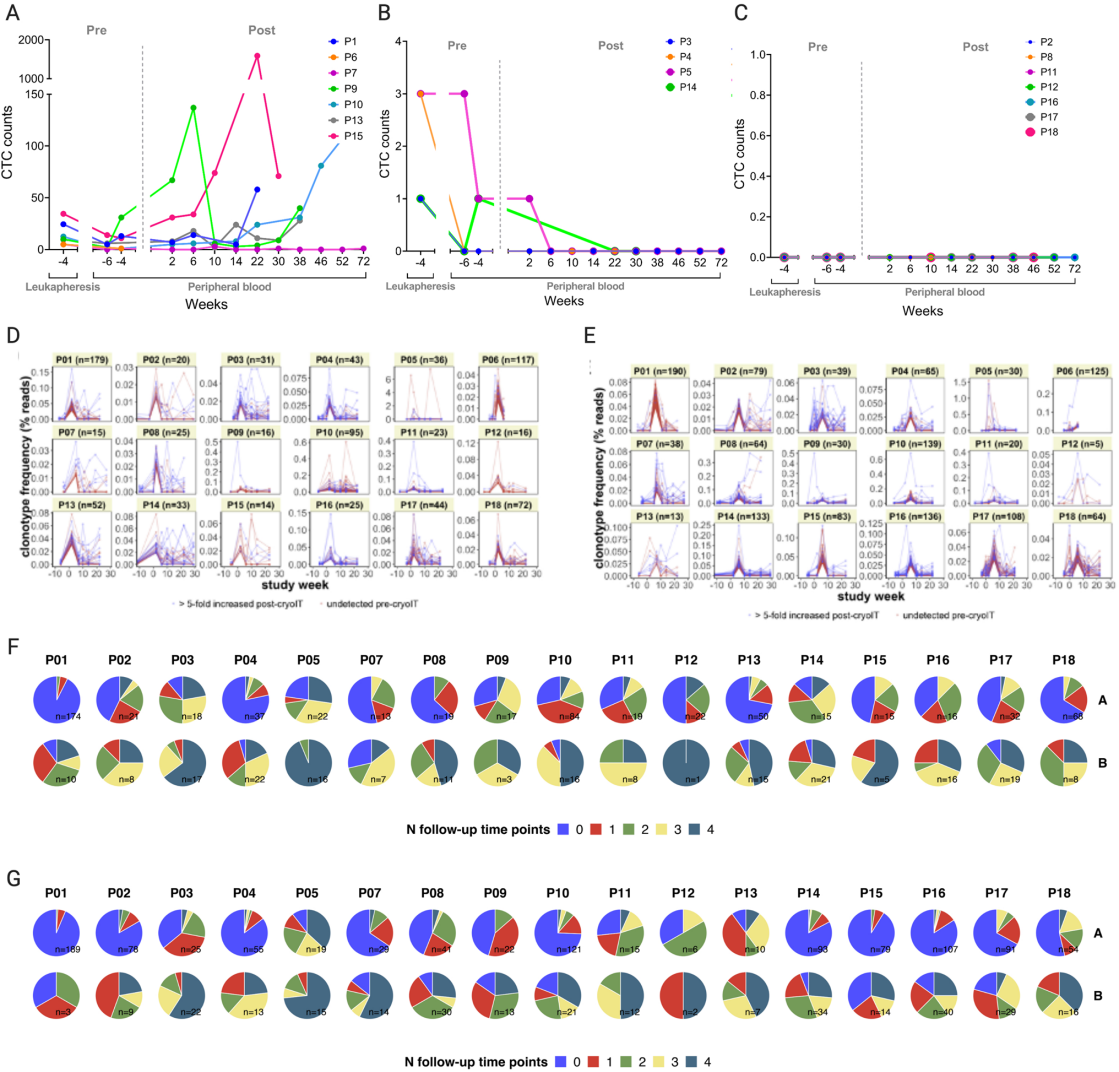
Changes in circulating tumor cells (CTC) and T cell receptor (TCR) clonotypes. **A–C** CellSearch platform numbers of CTC prior to cryoimmunotherapy (CryoIT) in 7.5 ml peripheral blood and in the leukapheresis sample are shown along the Y-axis. Weeks pre-CryoIT (-) and post-CryoIT are shown on the X-axis. Participants are grouped according to pre-treatment levels of CTC: **A** CTC ≥ 5, **B** CTC = 1–4, and **(C)** No detectable CTC. The vertical dashed line shows the time of CryoIT. **D–E** Frequency of clonotypes over the course of the clinical trial up to 30 weeks following CryoIT. Frequency changes to the top 200 largest (number of sequence counts) clonotypes at either **D** two weeks or **E** six weeks after CryoIT were examined. The individual patient graphs show how many (*n*) of the 200 largest clonotypes which were either undetectable prior to treatment (red) or > fivefold expanded after the CryoIT (blue). For the pretreatment time points, maximum clonal frequencies were used. Time in weeks is shown on the x-axis, with 0 indicating time of the CryoIT. Plots **F** and **G** show the longevity of clonotypes identified in the samples collected two **F** and six **G** weeks after the CryoIT. Pie charts are colored according to total number of follow-up time points at which the clonotypes in the samples were identified. Zero (blue) indicates clonotypes which were not detected in the samples at any of the four time points subsequent to either week 2 or 6 post-CryoIT. Group A: Clonotypes that were undetectable in all available pre-CryoIT samples; Group B: Clonotypes which were at least fivefold expanded compared to available pre-CryoIT samples

All patients with pre-treatment CTCs showed either transient CTC decreases which lasted months for several patients (pre-treatment CTC ≥ 5/7.5, *n* = 7), or complete CTC disappearance (pre-treatment CTC 1–4, *n* = 4). None without CTCs prior to CryoIT (*n* = 7) developed CTCs during follow-up (Fig. 3A-C, Supplementary Table S6).

Two weeks after the intervention, CTC numbers were available for 12/18 (67%). The CTC response analyses identified longer PFS for those with complete disappearance of CTCs (*p* = 0.016, Fig. 2D). When examining CTC responses in the first samples taken after the CryoIT (*n* = 16/18), similar results were seen (*p* = 0.0014, Supplementary Fig. S7), while those with CTC response = 0 demonstrated longer OS (*p* = 0.016).

**Fig. 4:**
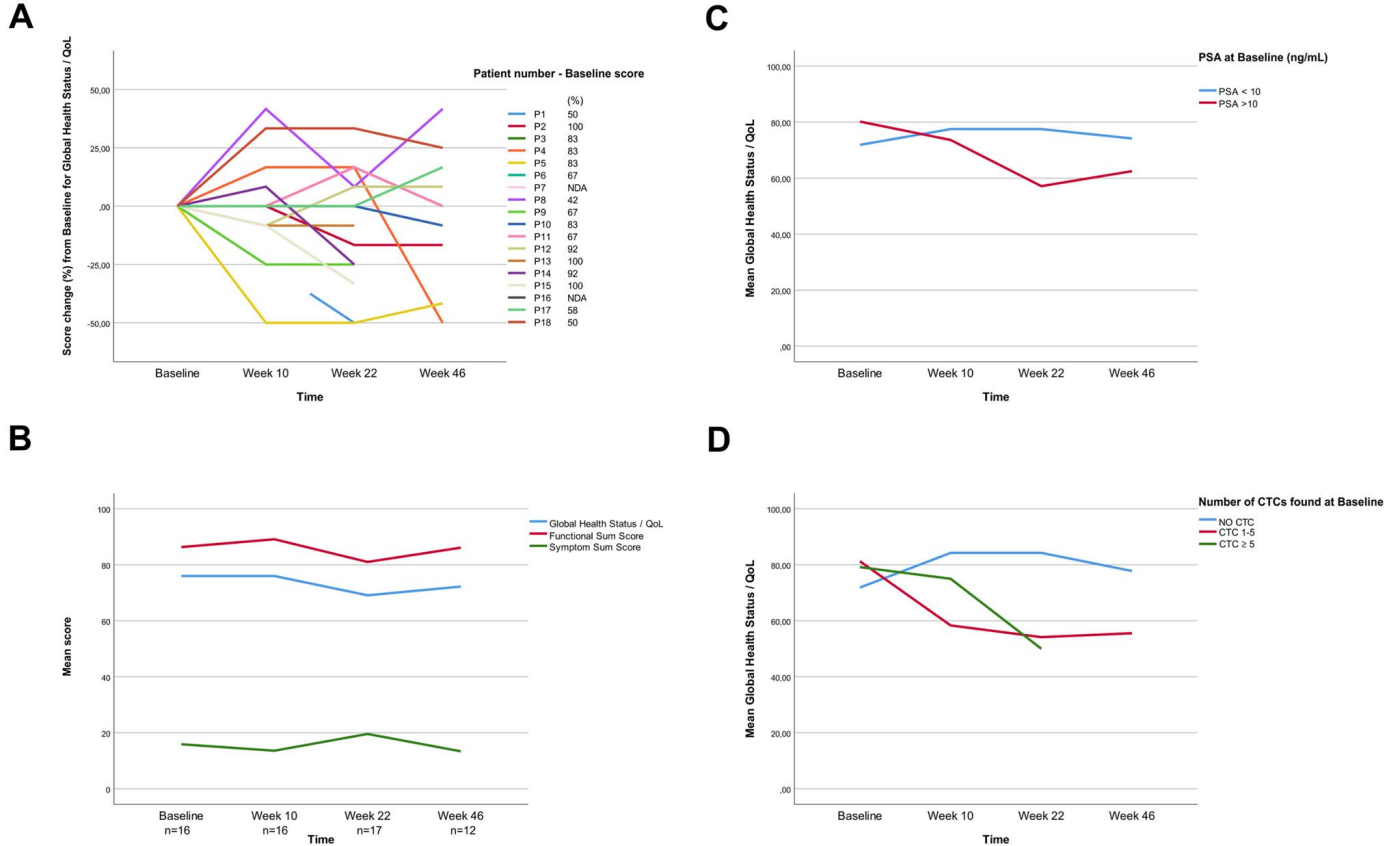
Health-related quality-of-life measurements. Time in weeks since trial inclusion (baseline) is illustrated along the x-axis of all plots. **A** Spider diagram illustrating the individual changes in the scoring of the Global Health Status/Quality of Life domain (questions 29–30 in the EORTC-QLQ-C30 questionnaire) over time. For each patient, the baseline score is presented next to the patient number. To the right, the individual pre-treatment (baseline) scores of the participants are given. **B** Line plot illustrating the overall stability of the functional and symptom sum scores, as well as the Global Health Status/Quality of Life. Numbers (*n*) indicate how many participants completed the EORTC-QLQ-C30 questionnaire at each time point. **C-D** Line plots demonstrating the effect on the Global Health Status/Quality of Life over time according to **C** the PSA-levels at baseline, and **D** the dichotomized presence or absence of circulating tumor cells (CTC) at baseline. QoL; Quality of Life

Figure 4 illustrates descriptively how the sequentially collected HRQoL scores were stable overall for the cohort. High PSA (> 10) and presence of CTCs prior to treatment were generally associated with worse HRQoL scores over time. Furthermore, pre-treatment CTCs associated with lower Global Health Status/HRQoL at week 22 (*p* = 0.03). Throughout the study, the VAS pain scores were stably low (mean 0.9–2.2) (Supplementary Fig. S3).

### Translational outcomes

While higher baseline ALP levels correlated with higher tissue expression of Tregs (*p* = 0.047) and higher ratios of FoxP3^+^/CD3^+^cells (*p* = 0.012) and FoxP3^+^/CD8^+^cells (*p* = 0.012) in the immunohistochemistry analyses of the tissue biopsies, lower tissue ratios of CD4^+^/CD3^+^cells associated with longer OS (*p* = 0.002) (Supplementary Figs. S4, S5, S6, Supplementary Tables S3, S4, S5). Treatment-related immunologic responses could not be identified by flow cytometry analyses.

TCR sequencing of blood samples revealed that the median number of novel and > fivefold expanded clonotypes was 35.5 (IQR 27.5–63.5) after two weeks, compared to 70.5 (IQR 37.0–125.25) six weeks after CryoIT (Fig. 3D-G, Supplementary S11, Supplementary Fig. S8). A median of 28% (IQR 17.55–41.48%) of the 200 most prevalent clonotypes was found both two and six weeks after treatment, while 36% (IQR 29.09–40.94%) was exclusive to each time point (Supplementary Table S7, Supplementary Fig. S9). Generally, the expanded clonotypes demonstrated greater longevity (≥ 20 weeks) compared to clonotypes undetectable prior to treatment (Fig. 3D-G).

While TCR clonotypes found in pre-treatment tumor biopsies could be identified among the clones detected in blood sampled pre- and post-treatment, less than 1% of the newly identified or expanded clonotypes was detected in the biopsy samples (Supplementary Table S8).

**Fig. 5:**
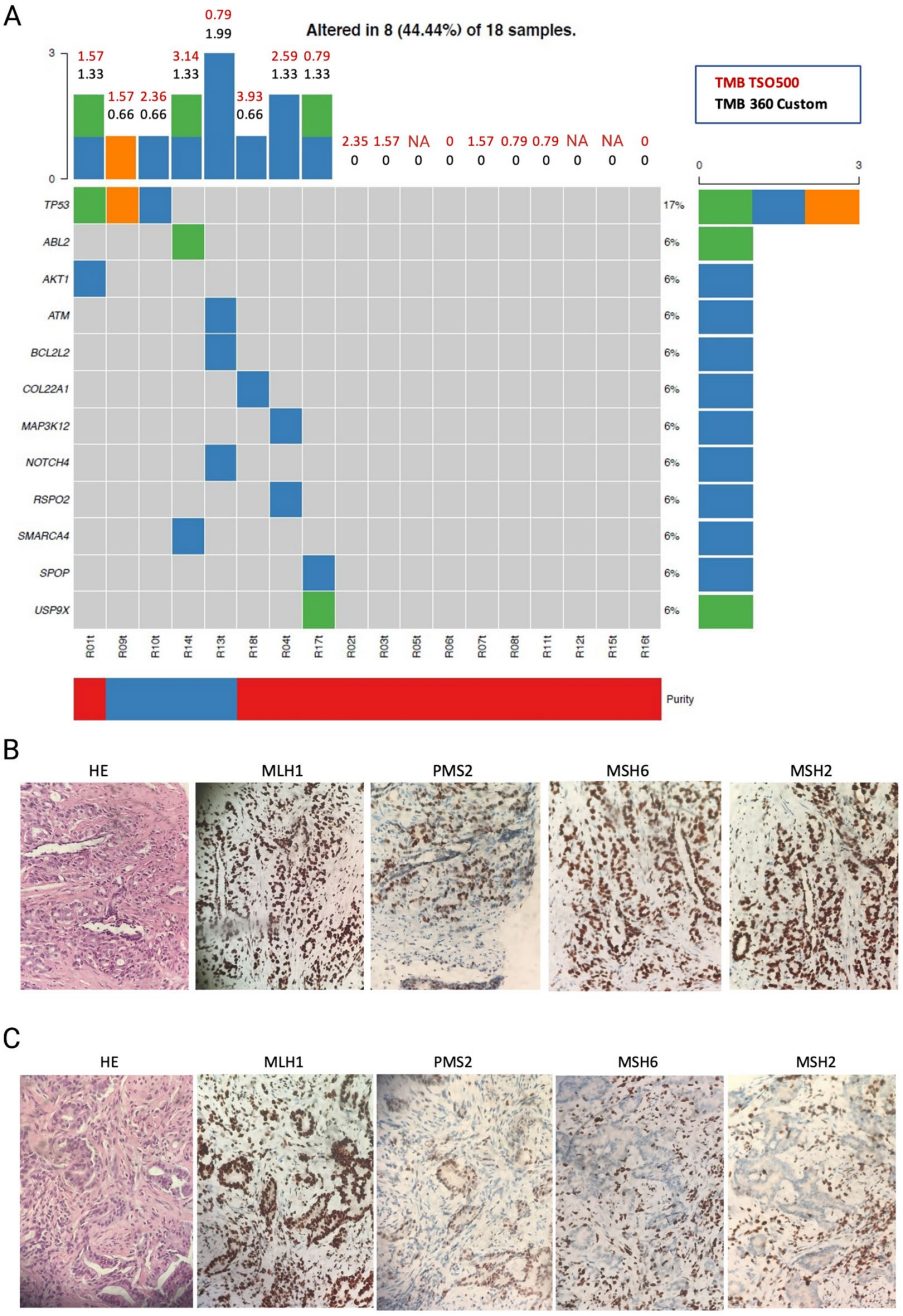
Results of the genetic and protein expression analyses of the tumor tissues. **A** The mutational landscape of 18 mCRPC tumor samples. Mutation distribution as detected by targeted sequencing of 360 cancer related genes, plotted as mutation per gene (rows) among the 18 patients (columns). Green, orange, and blue illustrate nonsense mutations, frameshift mutations, and missense mutations, respectively. Numbers listed above the gray area are estimates of tumor mutational burden (TMB) per sample, where black numbers indicate TMB based on results from the 360-gene custom panel and red numbers indicate TMB estimated form a similar analysis based on Illumina TSO500. Bars and percentages to the right of the gray panel represent mutation frequency per gene. The bar under the gray plot area indicates the tumor cell fraction (TCF) as above 20% (blue) or below 20% (red) as estimated by the FACETS algorithm. **B-C** Microscopy images of the immunohistochemical protein expression in a formalin-fixed paraffin-embedded sample of the four MMR proteins examined for estimation of cancer cell microsatellite instability: MLH1, PMS2, MSH2, and MSH6. For tissue orientation, hematoxylin–eosin (HE) staining was performed on the first representative slides from each patient biopsy sample. **B** Sample staining pattern with > 10% positive expression of MMR proteins, representative for 17/18 participants. **C** Staining pattern for the one sample with equivocal results demonstrating < 10% positivity of protein expression of MSH6 and MSH2 in the tumor cell nuclei. NA; missing

Both the custom 360 gene and the TSO500 gene panels identified the tumors as MSS with low TMB, and immunohistochemical analyses of MSI status by MMR proteins confirmed MSS results. Most biopsies demonstrated low tumor cell fractions (< 20% cancer cells) (Fig. 5A and Supplementary S10). The TMB spanned 0–1.5 mutations/Mb (360 custom gene panel) and 0.79–3.93 mutations/Mb (TSO500 panel) (Fig. 5A). While eight patients demonstrated at least one mutated gene, only *TP53* was mutated among three samples, while the remaining twelve involved genes were unique to one patient.

## Discussion

In this pioneering mCRPC clinical trial, CryoIT was found to be safe and tolerable. This is consistent with the well-defined safety profile of cryoablation in solid tumors [19, 20, 31]. Furthermore, the maximum tolerated dose was not reached at doses of 2.0 × 10^8^ iDCs, which is in accordance with earlier reports stating that DC therapy is well tolerated [14, 15]. Urinary retention, previously associated with cryotherapy, was the most commonly reported AE. The likely cause was local tissue inflammation and subsequent edema resulting from cryoablation. No Suspected Unexpected Serious Adverse Reactions were registered, and most of the observed treatment-related AEs were grade 1–2. Moreover, the addition of checkpoint inhibitors neither altered the severity of registered AEs nor induced AEs previously reported for ipilimumab and pembrolizumab. After 46 weeks, 33% of participants had clinical benefit of the treatment according to all radiological evaluations. Most trials utilize either a combination of MRI and bone scans, or CT imaging. Since this trial commenced, the PCWG published updated prostate cancer trials guidelines on how to interpret bone lesions[32], but despite existing guidelines, no gold standard is yet established for radiological evaluation of immunotherapy in prostate cancer [20, 33].

For the total cohort, PFS was 10.5 months while median OS was 40.7 months. In this early phase trial, no controls were included. In a study including mCRPC patients with diverse risk profiles, median PFS were 11.1 months (IQR 3.7–21.8) versus 8.2 months (3.5–16.6) in the less detrimental and more disadvantageous group, respectively, while corresponding OS were 41.8 (23.4–53.6) and 28.4 (17.9–41.9) months [34]. Similar results have been demonstrated in other trials [35, 36]. The DC-based vaccine, sipuleucel-T, approved by the FDA for asymptomatic or minimally symptomatic mCRPC, has shown OS benefit to patients in three double-blind randomized phase III clinical trials. Median survival times were 25.8, 25.9, and 19 months for sipuleucel-T treated patients compared to 21.7, 21.4, and 15.3 months for placebo-treated patients [3, 37]. Our trial cohort ranged across risk groups, and the survival data are non-inferior to placebo cohorts from other trials.

The associations discovered in this trial between CTC response and PFS in prostate cancer correspond with previous findings in phase III trials, where either conversion from CTC counts ≥ 5 to CTC < 5 or total disappearance indicated favorable responses to therapy [38–40]. The transient decreases in CTC counts seen after the CryoIT in patients with high pre-treatment CTC (≥ 5) could suggest treatment-related effects.

The high proportion of expanded or new TCR clonotypes in blood, both two and six weeks after CryoIT, and the minimal intrapatient clonotype overlap could imply an treatment-related expansion or generation of TCR clonotypes with a time-dependent broadening of the peripheral clonotype repertoire. Several recent publications have found that expanded clonotypes in peripheral blood following immune checkpoint inhibition were different from pre-treatment tumor-infiltrating T-cell clonotypes and seemed to be replenished from outside of the tumor [41, 42].

Patients reported consistently high values in the functional and Global Health Status/HR-QoL domains and low values in symptom domains, but those with pre-treatment CTC reported a worse HRQoL over time. In contrast to other trials [11, 43, 44], we found no differences in HR-QoL at baseline between patients with clinical benefit and non-responders.

All the patients had MSS tumors with low TMB. This finding is in accord with previous studies identifying 1–2% of prostate cancers as MSI and/or TMB high [45, 46]. Still, there seem to be a prognostic and predictive role for TMB in the therapy with immune checkpoint inhibitor in mCRPC if the TMB ≥ 10 mut/Mb, as demonstrated in a comparative study between taxanes and immune checkpoint inhibitors for this patient group [47].

This work may be limited by a small sample size, and lack of sequential tumor biopsies and comparator groups. While utilization of low doses of cyclophosphamide could impact the CTC numbers, the doses applied are considerably lower than those required for a general cytotoxic effect. We applied imaging guidelines published prior to trial commencement in 2015. As guidelines were updated as new treatment and technologies developed during the trial period, the application of older versions could limit interpretation of radiologically based outcomes. Second line hormonal treatment and early chemotherapy was fully introduced in Norway during the enrolment period and time-to-event comparisons with historical controls will therefore be imprecise. Despite these limitations, the findings encourage a next phase CryoIT trial, powered to estimate potential treatment efficacy.

In conclusion**,** this first-in-class trial of CryoIT is safe and well tolerated for all iDC doses, also when combined with either a CTLA4 inhibitor or a PD-1 inhibitor. A third of the participants demonstrates durable clinical responses. Results indicate possible treatment-associated changes in the CTC levels and TCR repertoires. The combination of safety and evidence of biologic responses and immune activation encourage a CryoIT phase II trial.

### Supplementary Information

Below is the link to the electronic supplementary material.

Supplementary file1 (DOCX 14027 KB)

## Data Availability

The data from circulating tumor cell enumeration, TCR sequencing data from sorted T cells including the R code and flow cytometry data used during this study are available through the Alden Cancer Therapy II (kalland@uib.no) for non-commercial research purposes. The joint data access committee of Vestlandets Innovasjonsselskap AS and Alden Cancer Therapy II will grant data access and enter into appropriate data access agreement subject to any applicable ethical approvals upon review of project proposals.

## Abbreviations

AE: Adverse event
ALP: Alkaline phosphatase
CryoIT: Cryoimmunotherapy
CTC: Circulating tumor cells
CTLA-4: Cytotoxic T-lymphocyte protein 4
DC: Dendritic cell
ECOG: Eastern Cooperative Oncology Group Performance Status
EORTC: European Organization for Research and Treatment of Cancer
FDA: US Food and Drug Administration
GnRH: Gonadotropin-releasing hormone
HRQoL: Health-related quality of life
iDCs: Immature DCs
IQR: Interquartile ranges
ISUP: International Society of Urological Pathology
LDH: Lactate dehydrogenase
mCRPC: Metastatic castration-resistant prostate cancer
MSI: Microsatellite instability
MSS: Microsatellite stable
OS: Overall survival
PD-1: Programmed cell death protein 1
PFS: Progression-free survival
PSA: Prostate-specific antigen
TCR: T cell receptor
TMB: Tumor mutational burden
Treg: Regulatory T-lymphocyte
VAS: Visual Analogue Scale
95% CI: 95% Confidence intervals

## References

[1] Cornford P, Bellmunt J, Bolla M (2017). EAU-ESTRO-SIOG guidelines on prostate cancer part II: treatment of relapsing, metastatic, and castration-resistant prostate cancer. Eur Urol.

[2] Westdorp H, Creemers JHA, van Oort IM (2019). Blood-derived dendritic cell vaccinations induce immune responses that correlate with clinical outcome in patients with chemo-naive castration-resistant prostate cancer. J Immunother Cancer.

[3] Nuhn P, De Bono JS, Fizazi K (2019). Update on systemic prostate cancer therapies: management of metastatic castration-resistant prostate cancer in the era of precision oncology. Eur Urol.

[4] Kantoff PW, Higano CS, Shore ND (2010). Sipuleucel-T immunotherapy for castration-resistant prostate cancer. N Engl J Med.

[5] de Bono J, Mateo J, Fizazi K (2020). Olaparib for metastatic castration-resistant prostate cancer. N Engl J Med.

[6] Motzer RJ, Tannir NM, McDermott DF (2018). Nivolumab plus ipilimumab versus sunitinib in advanced renal-cell carcinoma. N Engl J Med.

[7] Larkin J, Chiarion-Sileni V, Gonzalez R (2015). Combined nivolumab and ipilimumab or monotherapy in untreated melanoma. N Engl J Med.

[8] Vitkin N, Nersesian S, Siemens DR, et al. The Tumour Immune Contexture of Prostate Cancer. Front Immunol. 2019;**10**(603) doi: 10.3389/fimmu.2019.00603. . 10.3389/fimmu.2019.00603 PMC6447686 30984182

[9] Slovin SF, Higano CS, Hamid O (2013). Ipilimumab alone or in combination with radiotherapy in metastatic castration-resistant prostate cancer: results from an open-label, multicenter phase I/II study. Ann Oncol.

[10] Kwon ED, Drake CG, Scher HI (2014). Ipilimumab versus placebo after radiotherapy in patients with metastatic castration-resistant prostate cancer that had progressed after docetaxel chemotherapy (CA184-043): a multicentre, randomised, double-blind, phase 3 trial. Lancet Oncol.

[11] Beer TM, Miller K, Tombal B (2017). The association between health-related quality-of-life scores and clinical outcomes in metastatic castration-resistant prostate cancer patients: exploratory analyses of AFFIRM and PREVAIL studies. Eur J Cancer.

[12] Sharma P, Pachynski RK, Narayan V (2020). Nivolumab plus ipilimumab for metastatic castration-resistant prostate cancer: preliminary analysis of patients in the CheckMate 650 trial. Cancer Cell.

[13] Fizazi K, Shore N, Tammela TL (2020). Nonmetastatic, castration-resistant prostate cancer and survival with darolutamide. N Engl J Med.

[14] de Vries IJ, Bernsen MR, Lesterhuis WJ (2005). Immunomonitoring tumor-specific T cells in delayed-type hypersensitivity skin biopsies after dendritic cell vaccination correlates with clinical outcome. J Clin Oncol.

[15] Banchereau J, Palucka AK, Dhodapkar M (2001). Immune and clinical responses in patients with metastatic melanoma to CD34(+) progenitor-derived dendritic cell vaccine. Can Res.

[16] Vignali DA, Collison LW, Workman CJ (2008). How regulatory T cells work. Nat Rev Immunol.

[17] Madondo MT, Quinn M, Plebanski M (2016). Low dose cyclophosphamide: mechanisms of T cell modulation. Cancer Treat Rev.

[18] Prise KM, O'Sullivan JM (2009). Radiation-induced bystander signalling in cancer therapy. Nat Rev Cancer.

[19] Oishi M, Gill IS, Ashrafi AN (2019). Primary whole-gland cryoablation for prostate cancer: biochemical failure and clinical recurrence at 5.6 years of follow-up. Eur Urol.

[20] Cookson MS, Aus G, Burnett AL (2007). Variation in the definition of biochemical recurrence in patients treated for localized prostate cancer: the American Urological Association Prostate Guidelines for Localized Prostate Cancer Update Panel report and recommendations for a standard in the reporting of surgical outcomes. J Urol.

[21] Aarts BM, Klompenhouwer EG, Rice SL (2019). Cryoablation and immunotherapy: an overview of evidence on its synergy. Insights Imaging.

[22] Le Tourneau C, Lee JJ, Siu LL (2009). Dose escalation methods in phase I cancer clinical trials. J Natl Cancer Inst.

[23] Farup PG, Skar V (2002). Collaboration by use of the Internet yields data of high quality and detects non-uniform management of patients with Helicobacter pylori infection. Scand J Gastroenterol.

[24] Eisenhauer EA, Therasse P, Bogaerts J (2009). New response evaluation criteria in solid tumours: revised RECIST guideline (version 1.1). Eur J Cancer.

[25] Scher HI, Halabi S, Tannock I (2008). Design and end points of clinical trials for patients with progressive prostate cancer and castrate levels of testosterone: recommendations of the Prostate Cancer Clinical Trials Working Group. J Clin Oncol.

[26] Aaronson NK, Ahmedzai S, Bergman B (1993). The European Organization for Research and Treatment of Cancer QLQ-C30: a quality-of-life instrument for use in international clinical trials in oncology. J Natl Cancer Inst.

[27] Yates LR, Gerstung M, Knappskog S (2015). Subclonal diversification of primary breast cancer revealed by multiregion sequencing. Nat Med.

[28] Raine KM, Hinton J, Butler AP (2015). cgpPindel: identifying somatically acquired insertion and deletion events from paired end sequencing. Curr Protoc Bioinform.

[29] Shen R, Seshan VE (2016). FACETS: allele-specific copy number and clonal heterogeneity analysis tool for high-throughput DNA sequencing. Nucleic Acids Res.

[30] Team RC (2020) R: A language and environment for statistical computing. R Foundation for Statistical Computing, Vienna, Austria

[31] Ramsay CR, Adewuyi TE, Gray J (2015). Ablative therapy for people with localised prostate cancer: a systematic review and economic evaluation. Health Technol Assess.

[32] Scher HI, Morris MJ, Stadler WM (2016). Trial design and objectives for castration-resistant prostate cancer: updated recommendations from the prostate cancer clinical trials working group 3. J Clin Oncol.

[33] Administration UFaD (2018) editor. Clinical Trial Imaging Endpoint Process StandardsGuidance for Industry

[34] Miller K, Carles J, Gschwend JE (2018). The phase 3 COU-AA-302 study of abiraterone acetate plus prednisone in men with chemotherapy-naive metastatic castration-resistant prostate cancer: stratified analysis based on pain, prostate-specific antigen, and gleason score. Eur Urol.

[35] Saad F, Fizazi K, Jinga V (2015). Orteronel plus prednisone in patients with chemotherapy-naive metastatic castration-resistant prostate cancer (ELM-PC 4): a double-blind, multicentre, phase 3, randomised, placebo-controlled trial. Lancet Oncol.

[36] Fizazi K, Jones R, Oudard S (2015). Phase III, randomized, double-blind, multicenter trial comparing orteronel (TAK-700) plus prednisone with placebo plus prednisone in patients with metastatic castration-resistant prostate cancer that has progressed during or after docetaxel-based therapy: ELM-PC 5. J Clin Oncol.

[37] Graff JN, Chamberlain ED (2015). Sipuleucel-T in the treatment of prostate cancer: an evidence-based review of its place in therapy. Core Evid.

[38] Watanabe H, Okada M, Kaji Y (2009). New response evaluation criteria in solid tumours-revised RECIST guideline (version 1.1). Gan to kagaku ryoho. Cancer Chemother.

[39] Scher HI, Armstrong AJ, Schonhoft JD (2021). Development and validation of circulating tumour cell enumeration (Epic Sciences) as a prognostic biomarker in men with metastatic castration-resistant prostate cancer. Eur J Cancer.

[40] Heller G, McCormack R, Kheoh T (2018). Circulating tumor cell number as a response measure of prolonged survival for metastatic castration-resistant prostate cancer: a comparison with prostate-specific antigen across five randomized phase III clinical trials. J Clin Oncol.

[41] Yost KE, Satpathy AT, Wells DK (2019). Clonal replacement of tumor-specific T cells following PD-1 blockade. Nat Med.

[42] Zhang J, Ji Z, Caushi JX (2020). Compartmental analysis of T-cell clonal dynamics as a function of pathologic response to neoadjuvant PD-1 blockade in resectable non-small cell lung cancer. Clin Cancer Res.

[43] Health USDo, Human Services FDACfDE, Research, et al. (2006) Guidance for industry: patient-reported outcome measures: use in medical product development to support labeling claims: draft guidance. Health Qual Life Outcomes. **4**:79 doi: 10.1186/1477-7525-4-79. 10.1186/1477-7525-4-79 PMC1629006 17034633

[44] Health USDo, Human Services FDACfDE, Research, et al. (2006) Guidance for industry: patient-reported outcome measures: use in medical product development to support labeling claims: draft guidance. Health Qual Life Outcomes. 79 10.1186/1477-7525-4-79 PMC1629006 17034633

[45] Baretti M, Le DT (2018). DNA mismatch repair in cancer. Pharmacol Ther.

[46] Le DT, Durham JN, Smith KN (2017). Mismatch repair deficiency predicts response of solid tumors to PD-1 blockade. Science.

[47] Graf RP, Fisher V, Weberpals J (2022). Comparative effectiveness of immune checkpoint inhibitors vs chemotherapy by tumor mutational burden in metastatic castration-resistant prostate cancer. JAMA Netw Open.

