# Supplementary material for: A phase I prospective, non-randomized trial of autologous dendritic cell-based cryoimmunotherapy in patients with metastatic castration-resistant prostate cancer
Source: Cancer Immunol Immunother. 2023 Mar 20;72(7):2357–73. doi: 10.1007/s00262-023-03421-7 (PMC10264291; doi:10.1007/s00262-023-03421-7)
Supplement: Supplementary file 1 — Supplementary file1 (DOCX 14027 KB) [file 262_2023_3421_MOESM1_ESM.docx]

**Supplementary Material**

Cancer Immunology, Immunotherapy (submitted in 2022) - Liv Cecilie V Thomsen, Alfred Honoré, et al.

**Table of contents**

|  | **Page** |
| --- | --- |
| **Supplementary S1** Demographics and clinical evaluations | **4** |
| A. Cohort characteristics | **4** |
| Supplementary Table S1 | **4** |
| Supplementary Figure S1 | **6** |
| B. Inclusion criteria | **7** |
| C. Exclusion criteria | **7** |
| D. Adverse events evaluation | **8** |
| E. Dose limiting toxicity | **8** |
| F. Trial design | **8** |
| Supplementary Figure S2 Trial flow chart | **9** |
| G. Statistics | **10** |
| **Supplementary S2** Autologous immature dendritic cells (iDC) generation and distribution | **10** |
| A. Autologous iDC production | **10** |
| B. Autologous iDC distribution | **11** |
| **Supplementary S3** Cryoablation | **12** |
| **Supplementary S4** Immune checkpoint inhibition | **12** |
| **Supplementary S5** Cyclophosphamide distribution | **12** |
| **Supplementary S6** Patient reported outcomes | **12** |
| Supplementary Figure S3 Visual Analogue Score (VAS) | **14** |
| **Supplementary S7** Flow cytometry analyses | **14** |
| **Supplementary S8** Prostate tissue evaluation | **15** |
| A. Histopathological analyses | **15** |
| Methods | **15** |
| Results | **15** |
| Supplementary Table S2 Histopathological characteristics | **15** |
| B. Immunohistochemical analyses | **16** |
| Methods | **16** |
| Results | **16** |
| Supplementary Figure S4 Histological images of CD3, CD4, CD8 and FoxP3  expression by immunohistochemistry | **18** |
| Supplementary Table S3 Associations between CD3, CD4, CD8, FoxP3, FoxP3 /  CD8 ratio, FoxP3/CD3 ratio, and clinico-pathological features | **19** |
| Supplementary Table S4 Associations between either clinical variables or blood  values and the tissue expression of CD3, CD4, CD8, and FoxP3 at inclusion | **21** |
| Supplementary Table S5 Associations between clinical variables or blood values and  tissue expression of CD3, CD4, CD8, and FoxP3 ratios at inclusion | **22** |
| Supplementary Figure S5 Survival estimates by the Kaplan Meier method | **23** |
| Supplementary Figure S6 Survival according to high vs. low ratio of cells expressing  FoxP3 / CD3 in prostate tissue biopsies acquired directly prior to the CryoIT  procedure | **24** |
| **Supplementary S9** Enumeration of circulating tumor cells | **25** |
| A. Methods | **25** |
| B. Results | **25** |
| Supplementary Figure S7 Survival according to the CTC response | **26** |
| Supplementary Table S6 CellSearch enumeration of circulating tumor cells in peripheral  and leukapheresis blood from all patients pre- and post-CryoIT | **27** |
| **Supplementary S10** Tissue biopsy DNA analyses | **28** |
| A. Immunohistochemical analyses of MSI status by mismatch repair proteins | **28** |
| B. Extraction and quantitation of DNA from prostate cancer core biopsies | **28** |
| C. DNA sequencing analyses by a 360 gene custom panel | **28** |
| D. Illumina Trusight Oncology 500 (TSO500) gene panel analyses | **29** |
| Library preparation | **29** |
| TMB and MSI analyses |  |
| **Supplementary S11** Ultradeep T cell receptor sequencing | **29** |
| A. Methods | **29** |
| B. Results | **29** |
| Supplementary Figure S8 New and expanded clonotypes detected two (A, B) and six (C,D)  weeks post-CryoIT | **32** |
| Supplementary Table S7 Percentage of pre-treatment prostate tissue TCR clones identified  in post-treatment blood samples | **33** |
| Supplementary Table S8 Percentage of unique and shared clonotypes between the largest  200 clonotypes at two time points: 2 weeks and 6 weeks after the CryoIT. | **33** |
| Supplementary Figure S9 Venn diagrams depicting clonotypic overlap. | **33** |
| **Supplementary S12** Changes in immunological and routine laboratory  variables | **35** |
| Supplementary Figure S10 Percent changes in the PSA levels from nadir | **35** |
| Supplementary Table S9 Differences between response groups in median intra-patient  blood value changes (%) after the CryoIT | **36** |
| **References** | **37** |

**Supplementary S1** Demographics and clinical evaluations

***A Cohort characteristics***

Additional therapies given prior to CryoIT were the antiandrogens bicalutamide and/or enzalutamide (n=3), the androgen-synthesis inhibitor abiraterone (n=1), taxanes (n=10), radiopharmaceuticals (n=2), and symptomatic radiation therapy (n=1).

**Supplementary Table S1** Demographic characteristics at baseline

|  | **Total cohort**  **n=18** | **Cohort part 1**  **n=9** | **Cohort part 2**  **n=9** | **Difference in medians **** | **Patients with clinical benefit***  **n=6** | **Non-responders***  **n=11** | **Difference in**  **medians **** |
| --- | --- | --- | --- | --- | --- | --- | --- |
| **Variable** | **Median (IQR)** | **Median (IQR)** | **Median (IQR)** | **P-value** | **Median (IQR)** | **Median (IQR)** | **P-value** |
| ECOG performance status | 0 (0-0) | 0 (0-0) | 0 (0-0) | 0·17 | 0 (0-0) | 0 (0-0) | 0.84 |
| Age (years) | 70 (62-74) | 69 (62-73) | 71 (63-74) | 0·96 | 67 (60-73) | 70 (65-75) | 0.61 |
| Weeks from diagnosis | 133 (62-217) | 67 (57-228) | 159 (118-183) | 0·23 | 133 (83- 170) | 118 (58-199) | 0.65 |
| Height | 179 (175-185) | 181 (177-184) | 177 (174-185) | 0.66 | 178 (174-184) | 178 (177-186) | 0.58 |
| Weight | 92 (79-96) | 81 (77-95) | 92 (90-102) | 0·20 | 86 (78-92) | 95 (80-96) | 0.42 |
| Body mass index (BMI) | 27 (26-30) | 26 (25-27) | 30 (27-32) | 0.02 | 27 (25-29) | 26 (26-29) | 0.88 |
| Prostate specific antigen (PSA) | 8·4 (4·7-39·4) | 18·8 (6·8-51·5) | 5·8 (4·6-14·8) | 0·34 | 3.3 (2.6-4.6) | 18.8 (8.4-57.8) | 0.0006 |
| Alkaline phosphatase (ALP) | 77 (66-100) | 98 (74-166) | 67 (66-79) | 0·27 | 68 (67-87) | 79 (59-171) | 0.76 |
| Lactate dehydrogenase (LDH) | 189 (174-195) | 192 (164-196) | 186 (176-194) | 0·02 | 169 (160-176) | 193 (188- 203) | 0.06 |
| C-reactive peptide (CRP) | 1 (1-4) | 1 (1-4) | 2 (1-4) | 0·85 | 3 (1-6) | 1 (1-4) | 0.52 |
| Platelets | 212 (175-228) | 195 (174-236) | 224 (198-227) | 0·79 | 211 (184-226) | 203 (161-232) | 0.76 |
| Hemoglobin | 13·8 (13·5-14·3) | 14·4 (13·7-14·8) | 13·6 (13·2-13·8) | 0·04 | 13.7 (13.6- 13.8) | 13.8 (13.3- 14.5) | 0.96 |
| Leukocytes (LPK) | 6·7 (5·0-8·3) | 8·0 (6·2-8·5) | 5·0 (4·0-7·1) | 0·02 | 6.5 (5.4-8.2) | 7.1 (5.1- 8.2) | 0.88 |
| Neutrophils | 4·2 (2·8-5·1) | 4·8 (3·7-5·2) | 2·8 (2·1-4·9) | 0·06 | 4.1 (3.0-4.9) | 4.8 (3.2-5.2) | 0.69 |
| Lymphocytes | 1·7 (1·4-2·2) | 1·8 (1·6-2·4) | 1·5 (1·4-1·7) | 0·06 | 1.6 (1.5-2.4) | 1.7 (1.5- 2.1) | 0.96 |
| Monocytes | 0·48 (0·40- 0·68) | 0·66 (0·45-0·71) | 0·43 (0·35-0·50) | 0·11 | 0·53 (0.44- 0.69) | 0.50 (0.41- 0.67) | 0.73 |
| Basophils | 0·0 (0·0-0·1) | 0·0 (0·0-0·1) | 0·0 (0·0-0·0) | 0·13 | 0.0 (0.0-0.0) | 0·00 (0·00- 0·10) | 0.07 |
| Eosinophils | 0·1 (0·0-0·1) | 0·1 (0·0-0·2) | 0·1 (0·1-0·1) | 0·72 | 0.10 (0.10- 0.18) | 0·10 (0·00- 0·10) | 0.18 |
| Ratio CD4 positive / CD8  positive lymphocytes | 2·41 (1·58- 3·57) | 1·82 (1·34-7·10) | 2·42 (1·77-2·90) | 0·80 | 2.1 (11.5- 2.4) | 2.7 (1.6- 7.0) | 0.46 |
|  | **Total cohort**  **n=18** | **Cohort part 1**  **n=9** | **Cohort part 2**  **n=9** | **Difference in medians **** | **Patients with clinical benefit*** | **Non-responders*** | **Difference in medians **** |
| **Variable** | **Median (IQR)** |  |  |  | **Median (IQR)** | **Median (IQR)** | **P-value** |
| CD3 positive leukocytes (%) | 78·2 (71·5- 83·4) | 80·2 (71·9-83·5) | 76·2 (71·3-83·1) | 0·93 | 82.4 (74.4- 86.5) | 75.1 (68.8-83.2) | 0.22 |
| CD3 positive leukocytes (no·) | 1293 (1137- 1502) | 1378 (1141-2069) | 1168 (963-1408) | 0·26 | 1383 (1260- 1675) | 1142 (1115-1468) | 0.22 |
| CD4 positive lymphocytes (%) | 51·3 (41·5- 58·9) | 52·7 (40·5-62·3) | 49·9 (44·1-57·5) | 1·00 | 49.7 (42.7- 55.6) | 52.7 (42.4-60.8) | 0.81 |
| CD4 positive lymphocytes (no·) | 895 (694- 1092) | 1056 (697-1074) | 777 (693-1098) | 0·60 | 930 (783- 1096) | 988 (695-1086) | 0.66 |
| CD8 positive lymphocytes (%) | 21·3 (15·8-31·7) | 30·3 (20·7-37·3) | 20·7 (19·0-26·1) | 0·80 | 25.5 (20.6- 32.0) | 21.9 (9.4-30.7) | 0.40 |
| CD8 positive lymphocytes (no·) | 423 (279-509) | 504 (172-841) | 406 (295-445) | 0·55 | 472 (361-509) | 406 (223-630) | 0.46 |
| Natural killer cells (%) | 8·7 (6·7-11·2) | 9·7 (8·2-10·7) | 8·0 (6·7-11·3) | 0·60 | 7.4 (5.4- 10.0) | 8.9 (7.5-10.6) | 0.40 |
| Natural killer cells (no·) | 147 (117-179) | 155 (150-202) | 125 (110-130) | 0·08 | 136 (118-169) | 153 (118-191) | 0.73 |
| Regulatory T lymphocytes (%) | 2·0 (0·7-2·7) | 2·2 (0·3-2·7) | 1·9 (0·8-2·3) | 0·93 | 1.7 (1.0-3.1) | 1.9 (0.5-2.4) | 0.62 |

* Response according to radiologic and PSA evaluation at week 42

** According to Mann-Whitney U test

Interquartile Ranges 1-3; IQR, Eastern Cooperative Oncology Group; ECOG

Comparison of the participants of the two trial parts and of response groups according to the radiologic and PSA evaluations 42 weeks after treatment. Cohort part 1 was treated by cryoablation and immature dendritic cell (iDC) therapy, while Cohort part 2 was treated with cryoablation and iDC therapy plus additional inhibition of either CTLA4 or PD-1 by ipilimumab or pembrolizumab, respectively.

**Supplementary Figure S1** Survival

**A.** Overall survival according to inclusion in the first or second part of the trial


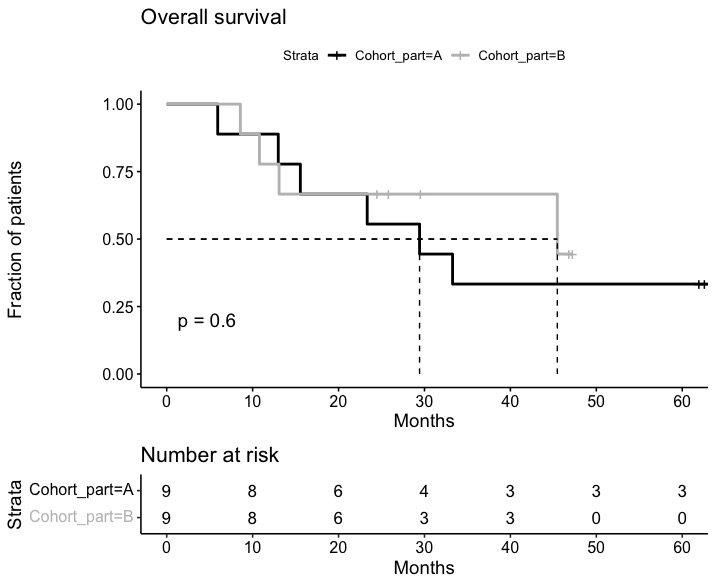


1st part

2nd part

**B.** Progression free survival according to the treatment received


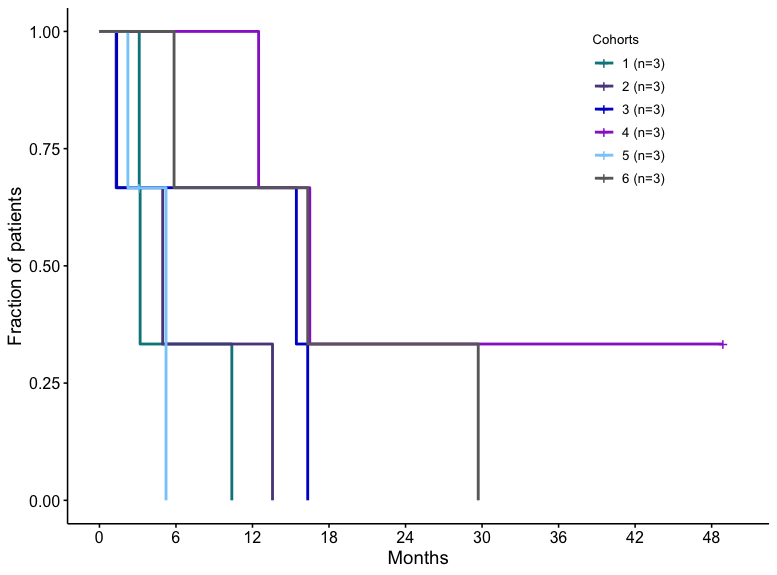


***B. Inclusion criteria***

All of the following conditions must apply to the prospective patient at screening prior to receiving a study agent:

| Castration resistant prostate cancer (CRPC) with radiologically proven metastasis beyond pelvic lymph nodes and chemotheraphy naïve **or** CRPC with radiologically proven metastasis beyond pelvic lymph nodes and chemotherapy finished more than three months earlier |
| --- |
| Must be ambulatory with an ECOG performance status of 0 or 1 |
| No contraindications for magnetic resonance imaging (MRI) (such as having pacemaker, claustrophobia, metal splints) |
| Must be able to undergo the surgical procedure under general or regional (spinal or epidural) anaesthesia |
| Must be at least 18 years of age |
| Must have lab values as follows:   - White Blood Cells ≥ 1.5 x 109/L - Platelets ≥ 100 x 109/L - Hemoglobin ≥ 9g/dL (≥ 5.6 mmol/L) - Creatinine ≤ 140 μmol/L; if creatinine is borderline, the creatinine clearance must be   ≥ 40 mL/min   - Bilirubin < 20% above the upper limit of normal - ASAT and ALAT ≤ 2.5 the upper limit of normal - Albumin ≥ 2.5 g/L - Serum-PSA < 200 ng/mL |
| Signed informed consent and expected cooperation of the patients for the treatment and follow up must be obtained and documented according to ICH/GCP and national and local regulations |

***C. Exclusion criteria***

Patients will be excluded from the study if they meet any of the following criteria:

| History of other prior malignancy, with the exception of curatively treated basal cell or squamous cell carcinoma of the skin or effectively treated malignancy that has been in remission for over 5 years and is highly likely to have been cured |
| --- |
| Treatment with any other investigational medicinal product within 4 weeks prior to first administration of the study drug |
| Adverse reactions to vaccines such as anaphylaxis or other serious reactions |
| History of immunodeficiency or autoimmune disease such as rheumatoid arthritis, systemic lupus erythematosus, sclerodermia, polymyositis-dermatomyositis, juvenile onset insulin-dependent diabetes, or a vasculitic syndrome |
| Significant cardiac or other medical illness that would limit activity or survival, such as severe congestive heart failure, unsTable angina, or serious cardiac arrhythmia |
| Active infection requiring antibiotic therapy |
| Known hypersensitivity to any of the components of the cell therapy product |
| Patients who test positive for hepatitis B, hepatitis C, or Human Immunodeficiency Virus (HIV) |
| Any other ongoing anti-tumour treatment administered, including chemotherapy, immunotherapy, cytokines, interferons, protease inhibitors or gene therapy. The use of gonadotropin-releasing hormone (GnRH)-agonist/antagonists with or without bicalutamide is accepTable. |
| Use of any of the following concomitant medications:   - chronic use of corticosteroids except for asthma inhalers/topical use - any agent with a known effect on the immune system, unless it is being given at dose levels that are not immunesuppressive, e.g. prednisone at 10 mg/day or less - any alternative and complementary drugs that may affect the immune system or be potentially harmful to patients participating in phase I studies |
| Any reason why, in the opinion of the investigator, the patient should not participate |

According to the protocolled inclusion criteria the participants had to have castration resistant prostate cancer with metastasis. They had to be either chemotheraphy naïve or, if chemotherapy was given, they had to have a three-month wash-out period prior to inclusion. The included patients must have progressed on antiandrogen treatment, but if they were still treated by antiandrogens at time of inclusion they could continue use of this treatment during the trial.

***D. Adverse events evaluation***

In this cryoimmunotherapy (CryoIT) trial the dose limiting toxicities for iDCs [ADC2014] alone or in combination with either ipilimumab or pembrolizumab were estimated at each visit throughout the study to establish the maximum tolerated dose (MTD) of the treatment given. Evidence of toxicity from the iDC treatment was additionally evaluated during the first 24 hours following the study procedure.

A commonly used definition of adverse events (AEs) is given by the National Cancer Institute Cancer Therapy Evaluation Programme as any unfavorable and unintended sign (including an abnormal laboratory finding), symptom or disease temporally associated with the use of a medical treatment or procedure regardless of whether it is considered related to the medical treatment or procedure”.^1^

AEs were recorded and their severity graded (from 1; mild, to 5; death) on the basis of the Common Terminology Criteria for Adverse Events (CTCAE) v3.0 with the main focus on possible effects falling within the following four broad classification categories: a) allergy / immunology, b) hematology/bone marrow function, c) metabolic function/laboratory measurements, and d) renal function/genitourinary symptoms. The relatedness of any registered adverse event (AE) to the study was evaluated by the urologist according to the degree of attribution to the study drug(s) as well as the likeliness of the AE resulting from study participation. Five degrees of relation were listed, ranging from definitely related to unrelated. All AEs were documented according to protocol, and the sponsors were notified by the investigators within 48 hours of any severe adverse event (SAE) being reported, defined as AEs ≥ grade 3.

***E. Dose limiting toxicity***

Toxicity was defined as dose limiting if patients experienced any persistent Grade 4 toxicity. Maximum tolerated dose was defined as the dose level below which dose limiting toxicities are seen in ≥1 of 3 subjects.

For this trial, the starting dose and escalation of iDCs were based on pre-clinical data. The selection of dose levels of the one given bolus dose of intratumoral ipilimumab and the twice distributed pembrolizumab (intravenously), were selected based on the FDA/EMA accepted treatment doses and available literature.

***F. Trial design***

The design of a 3+3 trial design can be described as follows: "The starting treatment dose administered to the first cohort is based on preclinical toxicological data. For the subsequent cohorts increasing dose levels are given, after a predefined dose-increasing regime. Another cohort of three patients can only be treated at the next dose level if none of the patients in the first/previous cohort experiences a DLT. Should any of the participants experience a However, if any of the three patients in a cohort experiences a DLT, three further patients will be included in that cohort and be treated at that dose level.

**Supplementary Figure S2** Trial flow chart

**G. Statistics**

In this phase I clinical trial, sample sizes were not established based on statistical methods. In each dosage group of escalation and extension parts, at least three patients were included. The aim of the expansion was to further evaluate the toxicity profile rather than formally demonstrate any efficacy endpoints. The database cut-off date was August 16th, 2019, when all participants had reached at least 22 weeks follow up for safety. PFS and OS were estimated with a snapshot taken on April 30, 2021.

All AEs potentially associated with CryoIT were categorized according to their nature and severity. Demographic data of the 18 enrolled patients were summarized using descriptive statistics.

Statistical significance was defined as p<0.05, although due to the small sample size and hypothesis-generating nature of the analyses, significant results should be interpreted with caution. Adjustments for multiple testing were not performed. Comparisons between the nine first and the nine last included participants were performed by two-sided Mann-Whitney U tests and presented as medians with interquartile ranges (IQR). Similarly, the intra-patient percentage changes in laboratory and immunologic values from pre-treatment values to results two and six weeks after therapy were compared between response groups by use of two-sided Mann-Whitney U tests. Results were listed as medians with IQR. Median time to progression, the PFS time period, and median OS with corresponding 95% confidence intervals (95% CI) were estimated by the Kaplan–Meier method and independent groups compared by log-rank tests. Percentage changes in PSA and LDH between pre-treatment values and samples taken two weeks after CryoIT were illustrated by waterfall plots, as were changes in the prostate cancer tissue size as measured on MRI at three sequential time points (week 14, 22 and 46 after CryoIT). For the results of the the CTC counts (Figure 3A-C) and the global HRQoL spider plot and line graphs (Figure 4) descriptive statistics were applied. Descriptive reporting was used to demonstrate changes in counts and longevity of the TCR clonotypes (Figure 3D-G)

The EORTC scoring manual, described in the Supplementary Information S6, was used For HRQoL analyses. Median VAS was calculated per patient per registration period before the means (with 95% CI) were plotted for the whole study population according to each period. Associations between HRQoL and CTC presence were made by the Mann-Whitney U tests with CTC as a dichotomous variable (absence/presence of CTC). Bioinformatic processing of the sequencing results (including alignment and quantification of TCR species) was performed by HS Diagnomics.

Data analyses were performed either in R v.3.6.0 or higher(61), with Excel v.16.21 (Microsoft), or by using IBM SPSS Statistics v.26 (IBM Corp.).

**Supplementary S2** Autologous immature dendritic cells (iDC) generation and distribution

***A. Autologous iDC production***

Leukapheresis was performed at Haukeland University Hospital 14 days prior to the cryoimmunotherapy using the Spectra Optia cell separator version 9 (Terumo BCT Inc., Lakewood, Colorado, USA), processing three total blood volumes of the autologous donor with the CMNC protocol. In the leukapheresis product collected the monocyte proportion should reach a minimum of 20 % and neutrophils a maximum of 2 % of total white blood cells. Thereafter the monocyte enrichment process (ELUTRATM System) took place, after the debulking of red blood cells where indicated. Cell suspensions enriched for monocytes were gravitationally transferred into a culture bag, where USP granulocyte-macrophage colony-stimulating factor (GM-CSF, Leukine®, Berlex, Richmond, CA) and USP interleukin 4 (IL4) were added to a final concentration in the media of 50 ng/mL and 20 ng/mL, respectively, after which the culture bag was incubated for 4 days in a 37 °C, 6% CO2 incubator.

Autologous immature dendritic cells were then produced at the Department of Cellular Therapy, Oslo University Hospital (the producer), according to the following protocol:

At arrival the cells were stored overnight. Monocytes were harvested from the apheresis product by elutriation and thereafter either frozen until later production or cultured fresh.

In the first 15 patients included in the study iDC were produced following a validated method that has been previously described.^2, 3^ Monocytes were differentiated into iDCs in a medium consisting of very low endotoxin (VLE)-Roswell Park Memorial Institute (RPMI) 1640 (Bio-chrome AG, Berlin, Germany) supplemented with 1,5% human serum AB (Institute für Transfusionmedizin Suhl, Suhl, Germany or PAN-Biotech GmbH, Aidenbach, Germany), GM-CSF (Leukine; Bayer HealthCare, Berlin, Germany), and IL-4 (R&D system, Minneapolis, Minnesota, USA). After 72 hours of incubation the iDC were washed and transferred into 20 vials, each containing 5x10^7^ iDC. The cells were cryopreserved in a cryo-solution containing 80% HSA (20%), 10% glucose (50%) and 10% DMSO at a total volume of 1000ul.

iDC of the last three patients were produced following an alternative validated and qualified production protocol as previously described.^4^ iDC were generated by culturing monocytes with CellGro DC-medium supplemented with granulocyte-macrophage colony-stimulating factor (GM-CSF) and interleukin (IL)-4 at a cell density of 1x10^6^ cells/mL for 5 days incubated at 37°C and with 5% CO_2_. Following the washing of cells, they were transferred into 20 vials with 5x10^7^ cells/vial and frozen using the same conditions as described earlier. All produced iDC were stored in a liquid nitrogen until use.

Vaccine production and quality control were performed according to Good Manufacturing Practice (GMP) in our GMP-facility at the Department of Cellular Therapy. Viability was assessed with trypan blue staining (Trypan Blue Staining Solution, ab233465) according to the European Pharmacopoeia (Eur.Ph) reference. Flow analysis was performed on the following markers: CD14, CD80, CD83, CD86, CD40, CCR7, HLA-DR, CD3, CD19, CD16+CD56, CD66b. Microbiological contamination was tested at the Department of Microbiology at The Norwegian Radium Hospital according to the Eur.Ph. The following relevant specifications had to be met for product release: viability >70% of total cells, purity >50% iDCs, and microbiological contamination negative. The iDCs were transported frozen by a courier to the study site for further use.

***B. Autologous iDC distribution***

On the day of treatment, all vials of autologous iDC were thawed within 2 hours of the immunotherapy procedure, then pelleted by centrifugation and resuspended in 1 ml of phosphate buffered saline (PBS). Cells were counted by a haemocytometer, and the correct number of iDC were transferred to a sterile syringe and brought directly to the operation theatre and injected directly into the cryoablated prostate tissue after the last thawing cycle and reconstitution of body temperature in the ablated tissue. iDC were administered in escalating dose levels, with the first three participants (n=3) receiving 5 x 10^7^ cells, the next three 1.0 x 10^8^ cells, and patients enrolled as numbers seven to nine receiving 2.0 x 10^8^ iDC. Since no dose-limiting toxicity was observed, all subjects in the expansion cohort (n=9) received the highest dose levels of about 2.0 x10^8^ cells.

**Supplementary S3** Cryoablation

One hour prior to the cryoablation procedure all patients received an intravenous dose of ciprofloxacin (400 mg/200 mL), and oral ciprofloxacin therapy was continued postoperatively, 500 mg twice daily, for eight days.

With the patient under general anesthesia and in the lithotomy position, cryoprobes and thermoprobes were placed transperineally into the prostate before a cystoscopy was performed. Thereafter, a urethral warming catheter and in-dwelling Foley catheter was placed through the urethra into the urinary bladder. Through utilization of a transrectal ultrasound, prostate measurements were noted and ultrasound scans compared to the preoperative MRI scans. Directly prior to the start of the first freezing cycle, eight tumor-targeted biopsies were obtained based on MRI cognitive fusion to preserve tissue for further study related analyses. Each biopsy was imediately diveded into two equal part. One part was formalin fixated for standard histopathology and immunohistochemistry analyses, while the other was snap frozen for later TCR sequencing and genomic analyses. Then the prostate was subjected to cryoablative temperatures at less than or equal to -40°C followed by warming to 37°C. Through cryoprobes freezing and warming was accomplished by pressurized Argon and Helium gases, respectively. Placement of the probes by the urologist was such that mainly the prostate tumors was frozen, not the adjacent tissues.

After the surgery a urethral catheter was inserted and initially left in place overnight. As the study progressed a decision was made to leave the catheter in place for one week for the last nine study participants due to the AE profiles identified in the nine participants being included in the escalation cohort.

**Supplementary S4** Immune checkpoint inhibition

Ipilimumab is a checkpoint inhibitor that blocks T-cell activity, including Treg activity, through the CTLA-4 pathway.^5^ Subjects enrolled in this study as participants 10-15 received ipilimumab 0.03 mg/kg (Yervoy^©^, Bristol-Myers Squibb) injected into the cryoablated area following injection of the iDC.

Pembroluzimab is a highly selective antibody for PD-1.^6^ In this study, the three subjects ultimately included received the checkpoint inhibitor intravenously as a single dose at 200 mg (Keytruda^©^, MSD Merck Sharp & Dohme B.V., Nederland) after the cryoimmunotherapy was performed.

**Supplementary S5** Cyclophosphamide distribution

Participants received low-dose cyclophosphamide as part of the study protocol to deplete the circulating regulatory T-lymphocyte population. Three days prior to surgery, all subjects had 300 mg/m2 cyclophosphamide infused intravenously. From week two after the cryoimmunotherapy procedure, all received cyclic cyclophosphamide every second week throughout the following 24 weeks; either 50 mg twice daily (the dose escalation cohort, n=9) or 50 mg once daily (the expansion cohort, n=9).

**Supplementary S6** Patient reported outcomes

**Method**

***Health-Related Quality of Life Inventory (EORTC-QLQ_C30)***

The Health-Related Quality of Life (HRQoL) was determined by employing the validated Norwegian translation of European Organization of Research and Treatment of Cancer (EORTC) QLQ-C30, version 3.0.^7^ On the scheduled visits (at baseline, and at week 10, 22, and 46 after treatment) the patients completed the EORTC-QLQ-C30 questionnaires. The study nurse gave paper versions of the inventory to the patients, who independently filled them out. After the patient had completed the form, the study nurse was available for questions. The results were manually entered into the study database.

Answers to the EORTC questions were given according to a four-point Likert format, with the exception of questions about general health and general quality of life, which were given according to a seven-point Likert format.

The indexes were scored according to the EORTC guidelines.^8^ The QLQ-C30 functional scales and the global scale were transformed so that 100% indicates the best function and 0% the lowest function of the individual HRQoL index. The QLQ-C30 symptom scales were transformed so that 0% indicated the least and 100% the most symptoms. Missing values were treated according to the EORTC QLQ-C30 scoring manual.^9^ The HRQoL scales consisting of more than one response were studied by Cronbach's α, with the general health/HRQoL scores compiled to one sum score.

Two additional sum scores were computed, compiling the functional indexes and the symptom indexes. This has previously been done in several studies, for instance by Aarstad^10-12^ and Hinz.^13^ Computing the score as a mean of the functional scales is a potential alternative of initial scoring to the EORTC-derived indexes. All sum scores, with the exception of nausea and vomiting, had a satisfactory Cronbach's α, thereby indicating it was psychometrically valid to calculate these indexes. Still, results of the sum scores must be interpreted in conjunction with the underlying indexes.

**Results**

Figure 4 illustrates descriptively how the HRQoL measurements is developing over the study period. While per patient analyses of EORTC-QLQ-C30 results visualized changes in reported HRQoL during follow-up, the HRQoL scores were sTable overall for the cohort. High PSA (>10) and presence of CTCs prior to treatment were generally associated with worse HRQoL scores over time.

When the total cohort was divided into two groups according to pre-treatment presence or absence of CTCs, statistical differences in the Global Health Status/HRQoL score were observed at week 22 (p=0.03). Regarding symptoms, changes over time were particularly evident in the fatigue and pain subdomains (not shown). Overall, the VAS pain scores were stably low (mean 0.9-2.2) throughout the study period (Supplementary Figure S3).

***Visual Analogue Scale (VAS)***

Median VAS was calculated per patient per registration period before the means (with 95% CI) were plotted for the whole study population according to each period.

**Supplementary S7** Flow cytometry analyses

Flow cytometry analyses of immune cells were conducted according to a local routine utilizing BD FACSCanto II (3 lasers) and BD FACSDiva software v.8.0.1 (BD Biosciences). The following cell subsets were identified by the flow cytometry analyses: the total T lymphocyte (CD3+ leukocytes) count and percentage of the total leukocyte number (CD45 high, low side scatter (SSC)), the CD4+ and the CD8+ as well as the double positive (CD4+CD8+) T lymphocyte count and percentage of the total T lymphocyte population, and the CD4+T lymphocyte /CD8+T lymphocyte ratio in the sample. Further, the regulatory T lymphocyte (TREGs; CD3+CD4+CD25+CD127-leukocytes) percentages of the CD4+T lymphocytes, the B lymphocyte (CD19+) and natural killer (NK) cell (CD56+) count and percentage of the total number of leukocytes were given.

**Supplementary S8** Prostate tissue evaluation

***A. Histopathological analyses***

***Methods***

Histopathologic evaluation of paraffin embedded tissues on hematoxylin and eosin stained slides from both primary diagnostic biopsies and prostate tissue biopsies directly prior to cryoablation. Dedicated uropathologists. re-examined the biopsies originally sampled from each participant at time of diagnosis to establish the primary diagnosis and to assess the histological type, International Society of Urological Pathology (ISUP) grade group, number of tumor-positive biopsies and the presence or absence of perineural invasion, extra-prostatic extension and vascular invasion. Study biopsies were assessed by immunohistochemistry for the presence of T cell subsets.

Hematoxylin and eosin stained slides were examined from the eight biopsies collected from each of the 18 patients. The one fresh frozen as well as the one paraffin embedded biopsy was with the most tumor tissue and the highest Gleason pattern was selected from each patient. The paraffin embedded samples were used for the immunohistochemistry analyses, while the evaluations by the two genetic panels and the TCR sequencing of tumor tissues were performed on the fresh frozen samples.

***Results***

**Supplementary Table S2** Histopathological characteristics

|  | **At diagnosis (n=18)** | **Prior to cryoablation (n=18)** |
| --- | --- | --- |
| ISUP grade group |  |  |
| 2 | 1 | 0 |
| 3 | 4 | 0 |
| 4 | 6 | 7 |
| 5 | 7 | 11 |
| Gleason pattern |  |  |
| 3+4 | 1 | 0 |
| 4+3 | 4 | 0 |
| 4+4 | 6 | 7 |
| 4+5 | 6 | 5 |
| 5+4 | 1 | 1 |
| 5+5 | 0 | 5 |
| Gleason score |  |  |
| 3-6 | 0 | 0 |
| 7 | 5 | 0 |
| 8 | 6 | 7 |
| 9 | 7 | 6 |
| 10 | 0 | 5 |
| Histological type |  |  |
| Acinar adenocarcinoma | 18 | 18 |
| Other | 0 | 0 |
| Perineural invasion |  |  |
| No | 0 | NA |
| Yes | 18 | NA |
| Extra-prostatic extension |  |  |
| No | 16 | NA |
| Yes | 2 | NA |
| Vascular invasion |  |  |
| No | 12 | NA |
| Yes | 6 | NA |

***B. Immunohistochemical analyses***

***Methods***

Hematoxylin and eosin (HE) stained slides from eight biopsies from each of the 18 patients were examined to assess histological type, ISUP grade group, number of tumor-positive biopsies and the number of inflammatory cells. Biopsies with the highest number of inflammatory cells (one biopsy per patient) were selected for immunohistochemistry staining with CD3, CD4, CD8 and FoxP3. Of the 18 selected biopsies, 16 contained tumor tissue.

*Immunohistochemistry*

CD3, CD4, CD8 and FoxP3 antibodies were stained using the platform Ventana BenchMark Ultra (Roche, F.Hoffmann-La Roche Ltd., Basel, Switzerland) and the detection system UltraView (CD3, CD8 and FoxP3) and OptiView (CD4). CD3 (Dako, A0452) was diluted 1:100 and incubated for 24 minutes. CD4 (CellMarque, SP35, 104R-16) was diluted 1:100 and incubated for 32 minutes. CD8 (Dako, C8/144B, M7103) was diluted 1:100 and incubated for 32 minutes. FoxP3 (BD Pharmingen, 259D/C7, 560044) was diluted 1:20 and incubated for 32 minutes. Positive and negative controls were included.

*Evaluation of staining*

CD3, CD4 and CD8 staining were predominantly detected in the cell membranes, whereas FoxP3 staining was detected in the nuclei.

The staining of all four antibodies were evaluated in a quantitative manner. For each staining, one area with the highest number of positive cells was selected (“hot spot”). In these hot spots, one area was counted in a high power field (HPF, x400) using an eye-piece graticule (10x10 gridlines; 0.25x0.25 mm; total 0.0625 mm^2^). The number of positive cells per area (0.0625 mm^2^) and the ratios between the four candidate markers per area were used for statistics. The upper quartile was used as a cut-off value for CD3, CD4, CD8, FoxP3, CD4/CD3 ratio and CD8/CD3 ratio, whereas the upper tertile was used as a cut-off value for the CD4/CD8 ratio, FoxP3/CD8 ratio and FoxP3/CD3 ratio. In survival analyses for overall survival, the upper quartile was used as a cut-off value for FoxP3/CD3.

*Statistics*

Associations between categorical variables were assessed by Fisher’s exact test. For continuous variables, the Mann-Whitney *U* or Kruskal-Wallis tests were used.

The Spearman rank correlation coefficient was used to determine the relationship between continuous variables. Univariate survival analyses were performed using the product-limit method (log-rank test) and Kaplan-Meier plots.

***Results***

The median CD3 count was 245.5 (min 59, max 789), the median CD4 count was 113.0 (min 7, max 367), and the median CD8 count was 80.0 (min 19, max 361), whereas the median FoxP3 count was 30.5 (min 2, max 189). In Figure S3 high and low counts of CD3, CD4, CD8, and FoxP3 are depicted.

As summarized in Table S3, high CD4 and FoxP3 counts and high FoxP3/CD8 and FoxP3/CD3 ratios were significantly associated with high ALP baseline level (p<0.047). High counts of CD3, CD4 and CD8 were significantly associated with low LD baseline level (p<0.046). High FoxP3/CD8 and FoxP3/CD3 ratios were borderline associated with shorter time since diagnosis at inclusion in the study (p=0.051 and p=0.062).

According to Fishers exact test, high counts of CD4 and FoxP3 and the high FoxP3/CD3 ratio were associated with shorter time since diagnosis at inclusion in the study (p<0.043) (Table S4 and Table S5).

A Spearman’s rank-order correlation was run to determine the relationship between the immunohistochemistry counts and ratios and the following variables: PSA prior to cryoimmunotherapy, ALP baseline level, LD baseline level and time from diagnosis to inclusion in the study. A significant or borderline positive correlation was found between ALP baseline level and the following: CD4 (rho=0.49 p=0.037), FoxP3 (rho=0.46, p=0.055), CD4/CD8 ratio (rho=0.46, p=0.058), FoxP3/CD8 ratio (rho=0.64, p=0.004), FoxP3/CD3 ratio (rho=0.57, p=0.014), and CD4/CD3 ratio (rho=0.45, p=0.060), whereas a strong negative correlation was found between CD3, CD4, CD8, and FoxP3 counts and LD baseline level (rho≤-0.51, p<0.042). FOXP3/CD8 ratio was negatively correlated with time since diagnosis at inclusion in the study (rho=-0.47, p=0.047), and a similar trend was found for FoxP3/CD3 ratio (rho=-0.45, p=0.059) regarding time since diagnosis. Further, the four candidate markers were significantly correlated with each other (rho=0.71-0.94, p≤0.001).

In univariate survival analyses, using progression-free survival and overall survival as end-points, high FoxP3/CD8 ratio was borderline associated with reduced progression-free survival (p=0.065). High FoxP3/CD3 ratio was marginally associated with reduced progression free survival (p=0.051). Finally, a high CD4/CD3 ratio was significantly associated with reduced overall survival (p=0.002) (Figure S4).

The cohort had aggressive cancer, with 13/18 (72%) classifying as ISUP grade group 4 or 5 at diagnosis, advancing to 18/18 (100%) at inclusion (Appendix S7). Patients with higher baseline serum-ALP demonstrated higher tissue expression of T regulatory cells (p=0.047) as well as higher ratios of FoxP3+/CD3+ (p=0.012) and FoxP3+/CD8+ cells (p=0.012). Results further indicated that patients with lower ratios of FoxP3+/CD3+ cells had longer PFS and that patients with lower ratios of CD4+/CD3+ had longer OS (Figure S7).

**Supplementary Figure S4** Histological images of CD3, CD4, CD8 and FoxP3 expression by immunohistochemistry

**
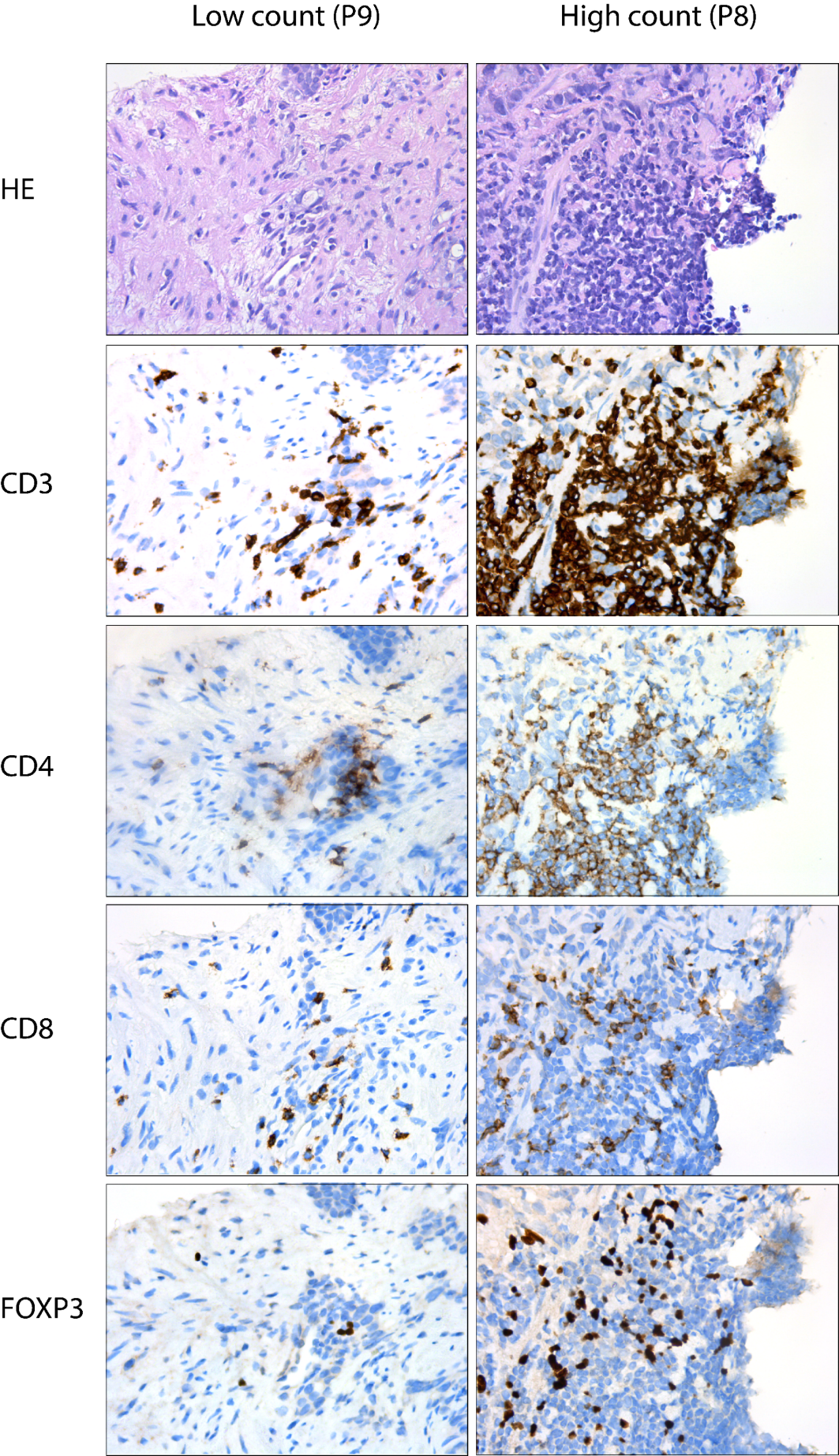
**

Original magnification x400

**Supplementary Table S3** Associations between CD3, CD4, CD8, FoxP3, FoxP3/CD8 ratio, FoxP3/CD3 ratio, and clinico-pathological features

|  | **CD3** | | | **CD4** | | | **CD8** | | | **FoxP3** | | | **FoxP3/CD8 ratio** | | | **FoxP3/CD3 ratio** | | |
| --- | --- | --- | --- | --- | --- | --- | --- | --- | --- | --- | --- | --- | --- | --- | --- | --- | --- | --- |
|  | Mean | Median | p-value | Mean | Median | p-value | Mean | Median | p-value | Mean | Median | p-value | Mean | Median | p-value | Mean | Median | p-value |
| ISUP grade group^1^  4  5 | 225.86  379.45 | 223.00  265.00 | 0.298 | 94.29  158.82 | 49.00  185.00 | 0.221 | 73.00  124.73 | 72.00  88.00 | 0.160 | 54.86  61.36 | 23.00  35.00 | 0.556 | 0.80  0.46 | 0.34  0.40 | 0.964 | 0.18  0.14 | 0.10  0.13 | 0.964 |
| Extraprostatic extension  Absent  Present | 303.69  448.00 | 245.50  448.00 | 0.623 | 134.00  131.50 | 113.00  131.50 | 0.779 | 92.63  200.50 | 80.00  200.50 | 0.574 | 55.88  82.50 | 30.50  82.50 | 0.574 | 0.61  0.45 | 0.36  0.45 | 0.574 | 0.16  0.19 | 0.12  0.19 | 0.399 |
| Vascular invasion  Absent  Present | 289.58  380.00 | 217.00  311.00 | 0.512 | 105.83  189.50 | 56.00  178.00 | 0.303 | 106.42  101.00 | 73.50  94.00 | 1.000 | 50.17  76.17 | 21.50  64.00 | 0.325 | 0.43  0.91 | 0.36  0.60 | 0.303 | 0.15  0.18 | 0.12  0.16 | 0.399 |
| PSA prior to CryoIT^2^  Low  High | 339.44  300.00 | 279.00  226.00 | 0.965 | 116.22  151.22 | 63.00  171.00 | 0.402 | 121.22  88.00 | 75.00  85.00 | 0.757 | 55.89  61.78 | 23.00  35.00 | 0.724 | 0.40  0.78 | 0.34  0.50 | 0.233 | 0.14  0.18 | 0.11  0.13 | 0.233 |
| Months since diagnosis at inclusion^2^  Low  High | 404.25  252.10 | 346.50  245.50 | 0.328 | 177.75  98.50 | 202.00  75.00 | 0.155 | 131.00  83.50 | 109.00  80.00 | 0.594 | 93.50  31.10 | 107.50  24.50 | 0.168 | 0.90  0.34 | 0.60  0.32 | 0.051 | 0.21  0.12 | 0.19  0.11 | 0.062 |
| ALP_Baseline^2^  Low  High | 240.44  399.00 | 226.00  290.00 | 0.145 | 87.81  179.56 | 26.00  171.00 | 0.047 | 75.56  133.67 | 75.00  138.00 | 0.270 | 25.33  92.33 | 23.00  75.00 | 0.047 | 0.32  0.86 | 0.31  0.54 | 0.012 | 0.10  0.22 | 0.10  0.19 | 0.012 |
| LD_Baseline^2^  Low  High | 498.13  179.25 | 527.00  193.50 | 0.012 | 201.00  85.88 | 221.00  58.50 | 0.046 | 159.88  60.63 | 158.50  49.50 | 0.016 | 96.88  32.25 | 96.50  23.00 | 0.059 | 0.60  0.67 | 0.46  0.37 | 0.345 | 0.18  0.16 | 0.16  0.12 | 0.674 |
| Treatment^3^  1  2  3  4  5  6 | 261.00  268.67  266.00  604.00  197.00  321.67 | 223.00  169.00  107.00  744.00  226.00  290.00 | 0.436 | 127.00  118.67  119.00  208.67  105.00  124.00 | 171.00  63.00  30.00  233.00  87.00  139.00 | 0.900 | 62.67  88.33  76.67  212.67  67.67  119.67 | 59.00  72.00  40.00  202.00  85.00  138.00 | 0.466 | 47.67  71.67  61.00  102.67  23.67  46.33 | 53.00  18.00  20.00  140.00  26.00  52.00 | 0.842 | 1.22  0.60  0.52  0.47  0.35  0.39 | 0.51  0.38  0.50  0.40  0.33  0.34 | 0.957 | 0.19  0.19  0.16  0.15  0.12  0.16 | 0.13  0.11  0.19  0.18  0.12  0.13 | 0.992 |
| Treatment^3^  1-3 and 5-6  4 | 262.87  604.00 | 223.00  744.00 | 0.038 | 118.73  208.67 | 87.00  233.00 | 0.260 | 83.00  212.67 | 72.00  202.00 | 0.066 | 50.07  102.67 | 26.00  140.00 | 0.213 | 0.61  0.47 | 0.38  0.40 | 0.678 | 0.16  0.15 | 0.13  0.18 | 0.953 |
| Cohort part  1 (escalation)  2 (expansion) | 265.22  374.22 | 169.00  279.00 | 0.310 | 121.56  145.89 | 63.00  139.00 | 0.757 | 75.89  133.33 | 59.00  88.00 | 0.171 | 60.11  57.56 | 20.00  35.00 | 0.724 | 0.78  0.40 | 0.50  0.34 | 0.627 | 0.18  0.14 | 0.13  0.13 | 0.757 |
| CTC present at baseline  No  Yes | 337.82  291.29 | 279.00  223.00 | 0.684 | 131.91  136.57 | 139.00  87.00 | 0.821 | 116.55  85.86 | 88.00  72.00 | 0.497 | 53.73  66.86 | 35.00  26.00 | 0.928 | 0.41  0.87 | 0.38  0.50 | 0.390 | 0.14  0.20 | 0.13  0.19 | 0.298 |
| CTC at baseline  None  1-5  >5 | 365.70  201.25  323.25 | 284.50  138.00  224.50 | 0.394 | 144.40  82.50  158.25 | 162.00  39.50  129.00 | 0.697 | 126.30  69.25  85.75 | 95.50  56.00  57.50 | 0.351 | 58.90  54.75  62.75 | 43.50  14.00  50.50 | 0.568 | 0.44  0.50  1.05 | 0.39  0.31  0.51 | 0.688 | 0.15  0.17  0.18 | 0.13  0.12  0.15 | 0.740 |
| Treatment response^4^  No  Yes | 276.75  405.67 | 224.50  431.50 | 0.574 | 132.33  136.50 | 113.00  117.50 | 0.779 | 86.33  141.17 | 78.50  123.00 | 0.574 | 55.33  65.83 | 30.50  37.50 | 1.000 | 0.68  0.40 | 0.45  0.32 | 0.303 | 0.17  0.13 | 0.13  0.11 | 0.223 |
| ^1^Gleason-grade in biopsies prior to cryoablation  ^2^Median as cut-off  ^3^ Treatment: 1) Dendritic cell therapy dose 1 , 2) Dendritic cell therapy dose 2, 3) Dendritic cell therapy dose 3, 4) Dendritic cell therapy dose 3 + ipilimumab dose 1, 5) Dendritic cell therapy dose 3 + ipilimumab dose 2, 6) Dendritic cell therapy dose 3 + pembrolizumab  ^4^Radiologically evaluated as patients with sustained sTable disease (PSSD) after 46 weeks | | | | | | | | | | | | | | | | | | |

**Supplementary Table S4** Associations between either clinical variables or blood values and the tissue expression of CD3, CD4, CD8, and FoxP3 at inclusion

|  |  | **CD3^1^** |  |  | **CD4^1^** |  |  | **CD8^1^** |  |  | **FoxP3^1^** |  |
| --- | --- | --- | --- | --- | --- | --- | --- | --- | --- | --- | --- | --- |
|  | Low | High | P value | Low | High | P value | Low | High | P value | Low | High | P value |
| Months since diagnosis at inclusion^2^ |  |  | 0.275 |  |  | 0.023 |  | 1 | 0.275 |  |  | 0.023 |
| Low | 5 | 3 |  | 4 | 4 |  | 5 | 3 |  | 4 | 4 |  |
| High | 9 | 1 |  | 10 | 0 |  | 9 | 1 |  | 10 | 0 |  |
| ALP_Baseline^2^  Low  High | 8  6 | 1  3 | 0.576 | 9  5 | 0  4 | 0.082 | 8  6 | 1  3 | 0.576 | 9  5 | 0  4 | 0.082 |
| LD_Baseline^2^  Low  High | 4  8 | 4  0 | 0.077 | 4  8 | 4  0 | 0.077 | 4  8 | 4  0 | 0.077 | 4  8 | 4  0 | 0.077 |
| Treatment response^3^  No  Yes | 11  3 | 1  3 | 0.083 | 10  4 | 2  2 | 0.569 | 11  3 | 1  3 | 0.083 | 10  4 | 2  2 | 0.569 |
| Months since diagnosis at inclusion^2^ |  |  | 0.275 |  |  | 0.023 |  | 1 | 0.275 |  |  | 0.023 |
| Low | 5 | 3 |  | 4 | 4 |  | 5 | 3 |  | 4 | 4 |  |
| High | 9 | 1 |  | 10 | 0 |  | 9 | 1 |  | 10 | 0 |  |
| ALP_Baseline^2^  Low  High | 8  6 | 1  3 | 0.576 | 9  5 | 0  4 | 0.082 | 8  6 | 1  3 | 0.576 | 9  5 | 0  4 | 0.082 |
| LD_Baseline^2^  Low  High | 4  8 | 4  0 | 0.077 | 4  8 | 4  0 | 0.077 | 4  8 | 4  0 | 0.077 | 4  8 | 4  0 | 0.077 |
| Treatment response^3^  No  Yes | 11  3 | 1  3 | 0.083 | 10  4 | 2  2 | 0.569 | 11  3 | 1  3 | 0.083 | 10  4 | 2  2 | 0.569 |
| Months since diagnosis at inclusion^2^ |  |  | 0.275 |  |  | 0.023 |  | 1 | 0.275 |  |  | 0.023 |

^1^Upper quartile as cut-off

^2^Median as cut-off

^3^Radiologically evaluated as patients with sustained sTable disease (PSSD) after 46 weeks

**Supplementary Table S5** Associations between clinical variables or blood values and tissue expression of CD3, CD4, CD8, and FoxP3 ratios at inclusion

|  |  |  | **CD4/CD8 ratio^1^** |  |  | **FoxP3/CD8 ratio^1^** |  |  | **FoxP3/CD3 ratio^1^** |  |  | **CD4/CD3 ratio^2^** |  |  | **CD8/CD3 ratio^2^** |  |
| --- | --- | --- | --- | --- | --- | --- | --- | --- | --- | --- | --- | --- | --- | --- | --- | --- |
|  |  | Low | High | P value | Low | High | P value | Low | High | P value | Low | High | P value | Low | High | P value |
| Months since diagnosis at inclusion^1^ |  |  |  |  |  |  |  |  |  |  |  |  |  |  |  |  |
|  | P value |  |  | 0.321 |  |  | 0.321 |  |  | 0.043 |  |  | 0.275 |  |  | 1.000 |
|  | Low | 4 | 4 |  | 4 | 4 |  | 3 | 5 |  | 5 | 3 |  | 6 | 2 |  |
|  | High | 8 | 2 |  | 8 | 2 |  | 9 | 1 |  | 9 | 1 |  | 8 | 2 |  |
| Baseline ALP^2^ |  |  |  |  |  |  |  |  |  |  |  |  |  |  |  |  |
|  | P value |  |  | 0.620 |  |  | 0.131 |  |  | 0.009 |  |  | 0.576 |  |  | 0.576 |
|  | Low | 7 | 2 |  | 8 | 1 |  | 9 | 0 |  | 8 | 1 |  | 8 | 1 |  |
|  | High | 5 | 4 |  | 4 | 5 |  | 3 | 6 |  | 6 | 3 |  | 6 | 3 |  |
| Treatment response^3^ |  |  |  |  |  |  |  |  |  |  |  |  |  |  |  |  |
|  | P value |  |  | 0.600 |  |  | 0.600 |  |  | 0.083^3^ |  |  | 1.000 |  |  | 0.569 |
|  | No | 7 | 5 |  | 7 | 5 |  | 1 | 11 |  | 9 | 3 |  | 10 | 2 |  |
|  | Yes | 5 | 1 |  | 5 | 1 |  | 3 | 3 |  | 5 | 1 |  | 4 | 2 |  |

^1^Upper tertile as cut off

^2^Upper quartile as cut off

^3^Lower quartile as cut off

^4^Median as cut-off

^5^Radiologically evaluated as patients with sustained sTable disease (PSSD) after 46 weeks

**Supplementary Figure S5** Survival estimates by the Kaplan-Meier method


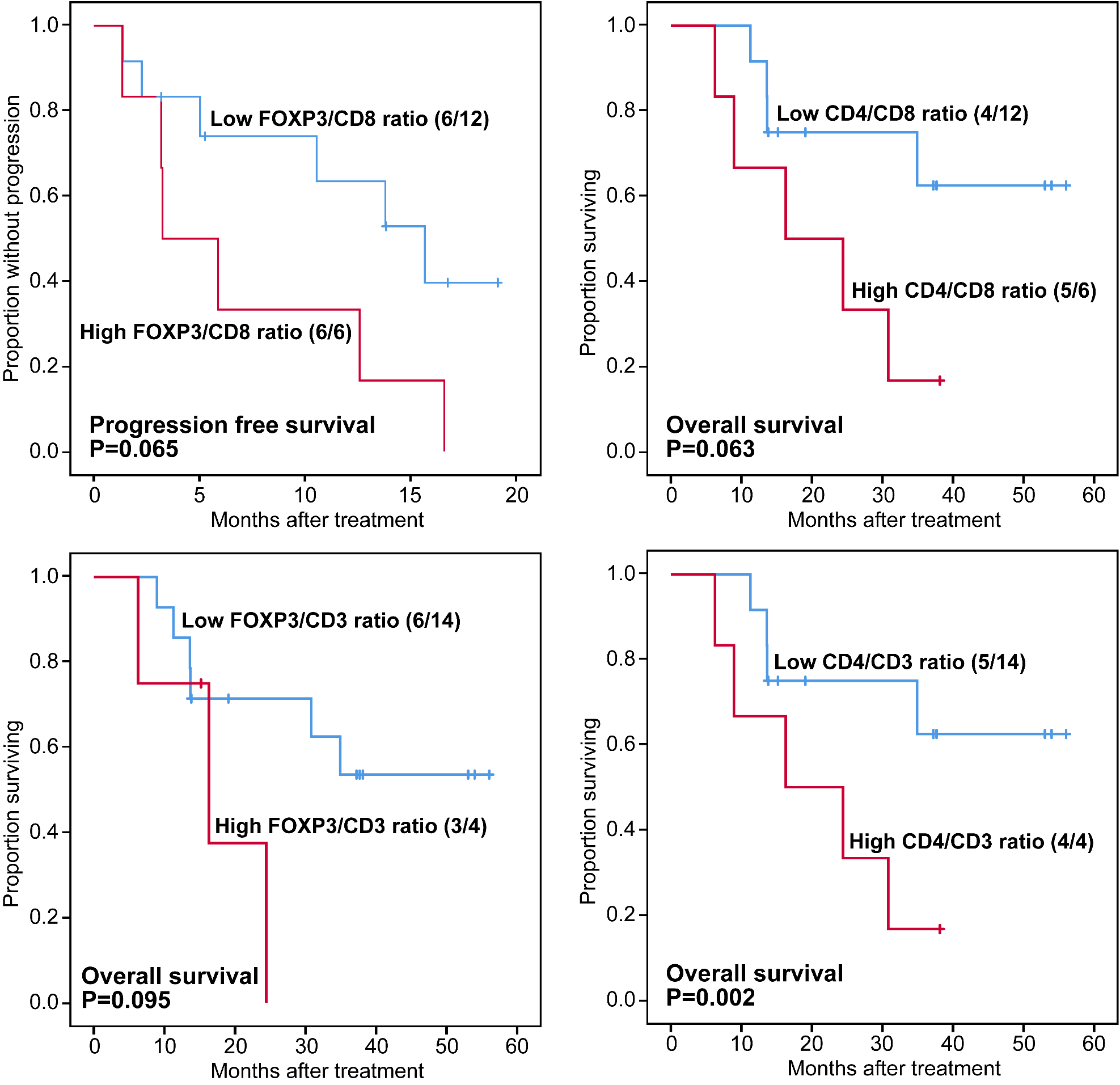


Estimates according to ratios of immune cells expressing CD3, CD4, CD8, and FoxP3 in prostate tissue prior to CryoIT treatment

**Supplementary Figure S6** Survival according to high vs. low ratio of cells expressing FoxP3/CD3 in prostate tissue biopsies acquired directly prior to the CryoIT procedure

**A.** Progression free survival

**
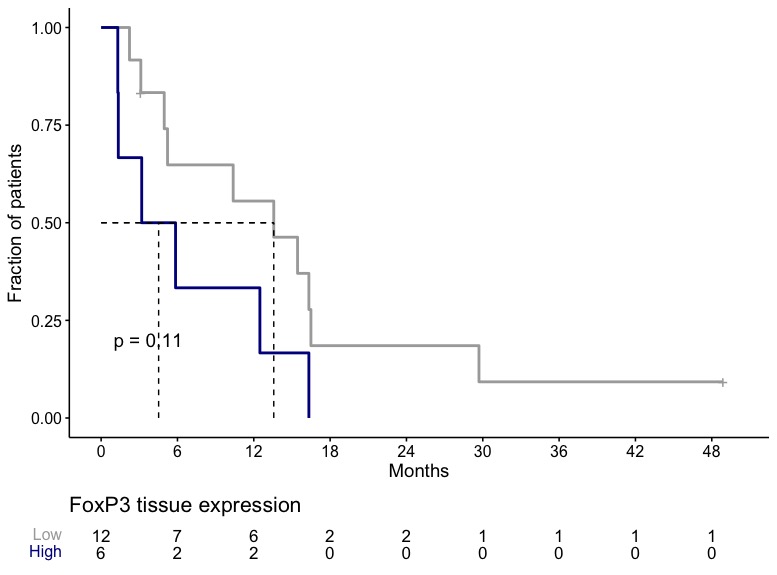
**

**B.** Overall survival

**
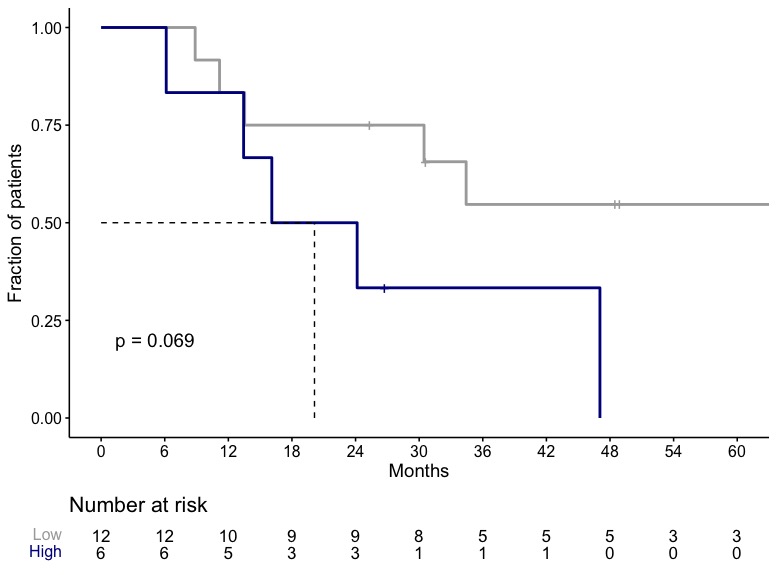
**

**Supplementary S9** Enumeration of circulating tumor cells

***A. Methods***

Peripheral blood or leukapheresis samples for enumerating circulating tumour cells (CTC) were collected in 7.5 ml CellSave tubes (Menarini Silicon Biosystems, Bologna, Italy). The samples were processed and analysed within 96 hours using the CellSearch™ assay (CellSearch™ Epithelial Cell Kit/CellSpotter™ Analyzer, Menarini Silicon Biosystems, Bologna, Italy) according to the manufacturer’s instructions. The CellSearch™ assay uses epithelial cell adhesion molecule (EpCAM)-based immunomagnetic enrichment and immunofluorescence with antibodies against keratins and CD45, differentiating between debris, leukocytes, and cells of epithelial origin. The assay provides high intra-observer, interobserver, and inter-instrument agreement and has been approved by FDA for use in prostate cancer patients.

Cells were defined as CTC if they were EpCAM-positive, labelled with the nuclear dye 4′,6-diamidino-2-phenylindole (DAPI), and immunostained with monoclonal keratin while negative for CD45-specific antibodies. The CellSpotter™ Analyzer was used by trained staff to detect CTCs as previously described.^14^

***B. Results***

The CTC enumeration revealed several trends. The data strongly confirmed that CTC counts above 5/7.5 mL blood is associated with worse prognosis. High CTC counts additionally predicted a limited effect of CryoIT and Supplementary treatments. Interestingly, patients with high CTC counts prior to CryoIT showed a transient decrease of CTCs, lasting up to several months following the treatment. When the participants eventually progressed according to radiological, laboratory and clinical data, increasing CTCs could be observed. Of patients without detectable CTCs prior to CryoIT in neither their leukapheresis product nor in their peripheral blood samples, none developed detectable CTCs in peripheral blood during the follow-up period of up to 72 weeks. Additionally, the subgroup of patients with CTC counts at 5 or less in repeated pre-treatment samples exhibited either loss of all CTCs or stabilized CTCs during the follow-up in the trial.

Due to the small number of patients these results can only suggest CryoIT effects. The results furthermore suggest that any CryoIT effects are more likely to occur if the treatment can be applied at less advanced stages of prostate cancer development, which would be feasible. In this CryoIT Phase I clinical trial, most of the participants have lived with their prostate cancer diagnosis for considerable time periods and were only recruited into the trial at the time when they already had developed metastatic disease and progressed on conventional androgen deprivation therapy (with increasing PSA/biochemical failure).

This clinical trial shows additional interesting results. Several of the patients with aggressive disease were treated with highly active androgen or androgen receptor repression when they progressed following CryoIT. According to PSA, ALP, LD, radiological, and CTC recordings, some exhibited strong treatment responses *e.g.,* patient P9. Consequently, one conclusion is that CryoIT does not preclude subsequent treatment effects of established therapy modalities. Whether synergy is reflected between highly active androgen repressive treatment and CryoIT remains uncertain, but this should be considered in the next generation protocols of CryoIT-based treatment of mCRPC.

**Supplementary Figure S7** Survival according to the CTC response. Survival depicted by Kaplan Meier plots. In all graphs months after the treatment is listed on the x-axis, while on the y-axis the fractions of patients alive, and for progression free survival plots; free of progression, are given. Survival have been analyzed according to the CTC response (as either no measurable or measurable CTCs after treatment). A) Overall survival according to CTC response 2 weeks after the CryoIT procedure. B) Overall survival according to the CTC response at the first time point after the CryoIT when CTCs were enumerated. C) Progression free survival according to the CTC response at the first time point after the treatment when CTCs were counted.

CTC; circulating tumor cells

**Supplementary Table S6** CellSearch enumeration of circulating tumor cells in peripheral and leukapheresis blood from all patients pre- and post-CryoIT

| **Patient** | **Leukapheresis** | | **Peripheral blood** | | **Peripheral blood** | | | | | | | | | |
| --- | --- | --- | --- | --- | --- | --- | --- | --- | --- | --- | --- | --- | --- | --- |
|  | **Pre-treatment** | | **Pre-treatment** | | **Weeks Post-CryoIT** | | | | | | | | | |
|  | Visit 2 (2 test tubes) | | Visit 1 | Visit 2 | 2 | 6 | 10 | 14 | 22 | 30 | 38 | 46 | 51/52 | 72 |
| P1 | 20 | 29 | 5 | 13 | 7 | 14 |  | 5 | 58 |  |  |  |  |  |
| P2 | 0 | 0 | 0 | 0 | 0 | 0 |  | 0 | 0 | 0 | 0 | 0 | 0 | 0 |
| P3 | 0 | 1 | 0 | 0 | 0 | 0 |  | 0 |  | 0 | 0 | 0 | 0 | 0 |
| P4 | 0 | 3 | 0 |  | 0 | 0 | 0 | 0 | 0 | 0 |  | 0 |  | 0 |
| P5 |  | 3 | 3 | 1 | 1 | 0 | 0 | 0 | 0 | 0 | 0 | 0 | 0 | 0 |
| P06 | 0 | 5 |  | 1 | NA |  |  |  |  |  |  |  |  |  |
| P07 | 0 | 5 | 0 | 1 | 0 | 0 | 3 | 0 | 0 | 1 | 0 | 0 | 0 | 1 |
| P08 | 0 | 0 | 0 | 0 | 0 | 0 | 0 |  | 0 | 0 |  | 0 |  |  |
| P09 |  | 10 | 2 | 31 | 67 | 137 | 6 | 3 | 4 | 9 | 40 |  |  |  |
| P10 | 10 | 15 | 0 | 1 | 5 | 6 |  | 8 | 24 |  | 31 | 81 | 107 |  |
| P11 | 0 | 0 | 0 | 0 |  |  |  |  | 0 |  |  | 0 |  |  |
| P12 | 0 | 0 | 0 | 0 | 0 |  | 0 | 0 | 0 |  | 0 | 0 | 0 |  |
| P13 | 5 | 14 | 6 |  | 8 | 18 | 2; 5 | 24 | 11 | 9 | 28 |  |  |  |
| P14 |  | 1 | 0 | 1 |  |  |  |  | 0 | 0 |  |  |  |  |
| P15 | 30 | 39 | 14 | 11 | 31 | 34 | 74 |  | 1589 | 71 |  |  |  |  |
| P16 | 0 | 0 | 0 | 0 |  |  |  |  |  |  | 0 |  | 0 | 0 |
| P17 | 0 | 0 | 0 | 0 |  |  |  | 0 | 0 |  | 0 |  | 0 |  |
| P18 | 0 | 0 | 0 | 0 |  |  | 0 |  |  |  | 0 | 0 |  |  |

Red color indicates samples collected prior to treatment: two tubes from the leukapheresis product and two samples from sequential visits.

All samples from peripheral blood collected after treatment are colored blue.

**Supplementary S10** Tissue biopsy DNA analyses

Since the microsatellite instability (MSI) of tumor cells and the mutational burden of solid tumors have been demonstrated to influence the responses to immunotherapy in other tumor types, these biomarkers were investigated in the biopsied tissues. The tumor in the prostate gland was biopsied directly prior to the cryoablative procedure.

**A. Immunohistochemical analyses of MSI status by mismatch repair proteins**

***Methods***

MSH2 (Ventana, RTU, 760-5093) was incubated for 32 minutes. MSH6 (Dako, M3646) was diluted 1:25 and incubated for 32 minutes. PMS2 (Ventana, RTU, 760-5094) was incubated for 32 minutes. MLH1 (Ventana, RTU, 760-5091) was incubated for 24 minutes. Positive and negative controls were included.

***Results***

The MSI analyses demonstrated intact protein expression (>10 % positive tumor nuclei) for MSH2, MSH6, PMS2 and MLH1 in 17 of the 18 cases (Figure 5B). One sample showed intact protein expression for PMS2 and MLH1, but an equivocal expression for MSH6 and MSH2 with <10 % positive tumor nuclei (Figure 5C). Overall, the protein expression analysis supported the fragment length analysis and did not indicate MSI of any of the biopsied tumors.

**B. Extraction and quantitation of DNA from prostate cancer core biopsies**

Prostate tumor tissue core biopsies (18 gauge) were obtained by transrectal ultrasound guided needle sampling. Each biopsy was cut in two halves. One half was quick-frozen and stored in liquid nitrogen and the other half was fixed in 4% formaldehyde in PBS followed by paraffin embedding (FFPE biopsies). The FFPE part was examined by trained pathologists to determine tumor contents and histologic variables. DNA were extracted from fresh frozen biopsies using Qiagen TissueRuptor and the Qiagen Allprep DNA/RNA Kit (Cat.no. 80004, Qiagen, Hilden, Germany) according to the manufacturer’s protocol. Yields of DNA of fresh frozen core biopsies were between 1,1 and 11,8 mg. DNA was extracted from 8 x 10 mm sections of 18 gauge FFPE core biopsies (QIAamp DNA FFPE Tissue kit, Cat.no 56404, Qiagen, Hilden, Germany) with yields between 48 and 164 ng. The Qubit 4.0 fluorometric HS assay was used for nucleic acids concentration in eluates (Qubit™ dsDNA HS Assay Kit, Thermofisher Scientific Cat. no. Q32851; Qubit™ RNA HS Assay Kit; Thermofisher Scientific Cat. no: Q32855). Biopsy materials were examined in parallel for microscopy of hematoxylin and eosin stained sections, immunohistochemistry, targeted DNA sequencing and T-cell receptor sequencing.

**C. DNA sequencing analyses by a 360 gene custom panel**

Targeted sequencing of 360 cancer related genes was used for assessment of mutations and copy number alterations. After filtering the patient specific copy number variants against those found in the patient blood samples, tumor cell ploidy was estimated. Due to a low tumor cell fraction in most of the biopsies, where cancer cells constitute <20% of the tissues, only four samples had high enough fractions to be included in the analyses. Of these, three demonstrated a tumor cell ploidy below 2, while one patient (P13) had an estimated ploidy of 3.29. For the patients included in the trial, the tumor mutational burden (TMB) spanned from 0 to 1.5 mutations/Mb, with eight of the patients demonstrating at least one mutated gene in the tumor tissue, involving a total of twelve genes. Of these mutated genes, only *TP53* was found to be mutated among three samples, while the other eleven gene mutations were unique to the specific patient sample.­ None of the patients had MSI tumors when the DNA-content extracted from the tumor tissue biopsies was evaluated by microsatellite fragment length analysis.

# **D. Illumina Trusight Oncology 500 (TSO500) gene panel analyses**

# ***Library preparation***

# MSI and TMB estimates were performed essentially as described (Kroeze LI The Journal of Molecular Diagnostics, Vol. 22, No. 6, June 2020, [doi.org/10.1016/j.jmoldx.2020.02.009](https://doi.org/10.1016/j.jmoldx.2020.02.009)). The Illumina TSO500 assay was used according to the manufacturer protocol with input of 40 – 100 ng nucleic acids. These analyses were performed by the Science for Life Laboratory, Uppsala University, Uppsala, Sweden.

***TMB and MSI analyses***

When the tumor biopsies were analyzed using the TSO500 gene panel, without filtering the mutational spectrum in the tumor against that of autologous blood samples, the TMB spanned from 0.79 to 3.93 mutations / Mb. MSI was not found for any of the patient biopsies according to TSO500.

**Supplementary S11** Ultradeep T cell receptor sequencing

***A. Methods***

T cell receptor (TCR) sequencing was performed using DNA isolated from peripheral blood mononuclear cells (PBMC) and Qiagen's AllPrep DNA/RNA Mini Kit (Qiagen, Hilden, Germany). T cell receptor beta (TRB) genes were amplified in a two-step PCR reaction including 44 TRB V-region (TRBV) and 14 TRB J-region (TRBJ) primers, with the addition of adapters necessary for the sequencing.^11^ Following quality control steps, sample libraries were sequenced on Illumina’s MiSeq instrument (Illumina, San Diego, CA, US), resulting in 2x150 base pair paired-end reads, which were processed to create Tables containing the clonotype information. Library preparation steps and sequencing, as well as the processing of the sequencing results, were performed by HS Diagnomics (Berlin, Germany). The resulting clonotype tables were analyzed using R (v3.6.0).^15^

In this analysis, new clonotypes were defined as not present at any of the pre-CryoIT time points. For expanded clonotypes, their frequency after CryoIT was required to be above a threshold of five times compared to the highest frequency of the respective clonotype among all available pre-CryoIT time points.

***B. Results***

From the participants, a total of 186 individual samples were available for the analysis of TRB sequencing of peripheral blood mononuclear cells. A median of 33196 clonotypes per patient sample (interquartile range (IQR) 25201-45408), and a total clonotype count median of 419638 (IQR 354681-463413) per sample, were identified. The total data set across all patient and time points contained over 5.9 million unique clonotypes, where clonotype uniqueness was set by TRB V-region, J-region and CDR3 nucleotide sequence information.

In order to identify clonotypes with potential anti-tumor activity, we focused on the following two time points: two and six weeks following CryoIT. PBMC samples were available at both of these defined study time points for almost all patients. In one case, for patient 7, the two-week time period was extended in order to include the corresponding sample in the analysis.

The top 200 clonotypes represented the largest clonotypes with respect to the number of TCR-sequencing reads within each clonotype. The top 200 clonotypes identified per person at the two time points were compared with the corresponding pre-treatment clonotypes found in the available pre-CryoIT samples and categorized according to two distinct criteria: clonotypes were either undetectable prior to treatment or they were at least 5-fold expanded at either week two or six after treatment (Figure 4 and Supplementary Figure S5).

The median number of clonotypes per patient that satisfied both criteria at 2 weeks post-CryoIT was 35.5 (IQR 27.5-63.5), compared to 70.5 (IQR 37.0-125.25) after six weeks. At week 2, these new and expanded clonotypes occupied a clonal space of 0.58% (IQR 0.38-1.62%) and 0.42% (IQR 0.16-0.63%), respectively, while occupying 1.42% (IQR 0.67-2.95%) and 0.51% (IQR 0.36-1.09%) of the clonal space at week 6 (Figure 3D-G, Supplementary Figure S8).

A total of eight clonotypes identified according to the aforementioned criteria were shared by different patients. An additional 16 and 23 clonotypes were shared between samples from week 2 and 6 post-CryoIT of individual patients, respectively, meaning that these clonotypes met the selection criteria in both weeks for a single patient. An important question regarding expanded or newly detected clonotypes was if these clonotypes remain detectable after CryoIT for durations longer than the two and six weeks.

Since TCR sequencing samples were not available for all patients over the entire period of the clinical study, the analysis was preformed based on four time points following week 2 and 6 post-CryoIT. All patients except patients P1 and P6 had four samples following week 6 available for this analysis. For patient P1, only three follow-up samples were available and included in this analysis. An insufficient number of follow-up samples led to the exclusion of patient P6. Three different scenarios were considered regarding the development of the clonotypes after their identification in week 2 or 6 post-CryoIT: (1) clonotypes were persistent during the entire follow-up period, which included four study time points and spanned until week 30 post-CryoIT; (2) clonotypes were lost within the follow-up period; or (3) clonotypes dropped below the detection limit at one to three of the follow-up time points, resembling a blip-like detection pattern.

In general, we observed clonotypes detected as >5-fold expanded at week 2 or 6 post-CryoIT as having a greater longevity compared to clonotypes that were undetectable at pre-CryoIT time points (Figure 4). A subgroup of clonotypes identified as >5-fold expanded at week 2 post-CryoIT was consistently detectable until at least 20 weeks after the treatment. The same observation was made for >5-fold expanded clonotypes at week 6 post-CryoIT for all but one patient (Supplementary Figure S6). For the groups with undetectable clonotypes prior to CryoIT, a consistent clonotype expression after two and six weeks post-treatment was seen for only 11 and 12 patients, respectively. Additionally, the frequency of long-lived clonotypes in the two 5-fold expanded groups was higher compared to the frequencies of durable clonotypes seen for the other two groups defined by novel post-treatment clonotypes (Supplementary Figure S6).

In addition to blood samples, TCR sequencing was also performed using DNA extracted from prostate cancer core biopsies obtained immediately prior to the CryoIT procedure. Only a small percentage of clonotypes detected in a patient throughout the study were detected in both blood and biopsy samples. While clonotypes resembling a pattern typical for expansion could be observed in a small number of patients, the clonotypes of most of the patients of this study did not allow such a conclusion. Thus, our current data set suggests that clonotypes defined as expanded are primarily ones that were either newly recruited or introduced by the CryoIT procedure itself.

Regarding clonotypes classified as expanded or new either two or six weeks after CryoIT treatment, the majority of clonotypes exclusively aligned with only one specific group. Only a small portion of clonotypes overlapped between week two and week six, when the threshold of the 200 clonotypes with the highest counts was applied. Within this overlap of clonotypes, those that were expanded after CryoIT treatment dominated. We created a series of Venn diagrams (below) to demonstrate the pattern of overlap as a quality control.

**Supplementary Figure S8** New and expanded clonotypes detected two (A, B) and six (C, D) weeks post-CryoIT. The clonotypes could either not be detected at any of the existing time points prior to CryoIT (A, C), or were 5-fold expanded compared to the existing pre-CryoIT time points (B, D). Grey-shaded areas of each graph illustrate the period after CryoIT in which 5-fold expanded or newly detected clonotypes were identified. Each clonotype is represented by a separate line, and the total number of clonotypes per sample (n) is shown in each graph.


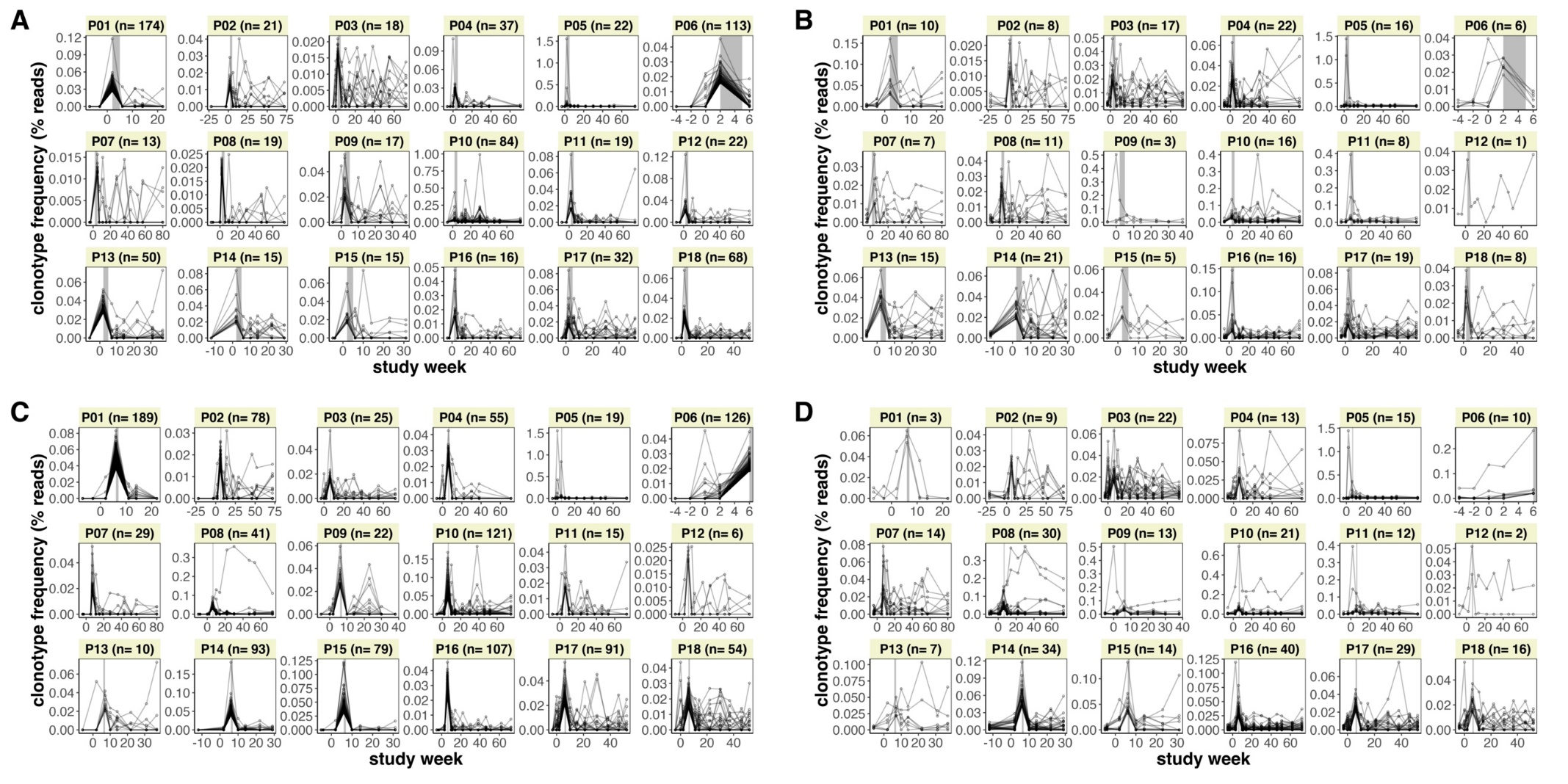


| **Supplementary Table S7** Percentage of pre-treatment prostate tissue TCR clones identified in post-treatment blood samples. Only the clonotypes identified as either new or at least 5-fold expanded after the CryoIT when compared to clonotypes in pre-treatment blood samples were included in the analyses. Clonotypes in post-treatment blood samples were examined 2 and 6 weeks after the CryoIT. | | | |
| --- | --- | --- | --- |
| **Identified clonotypes in the prostate tissue** | **IQR1** | **Median** | **IQR3** |
| Detected exclusively in the prostate tissue | 99.77 | 99.87 | 99.92 |
| New in blood after 2 weeks | 0.03 | 0.05 | 0.07 |
| New in blood after 6 weeks | 0.02 | 0.03 | 0.06 |
| Expanded in blood after 2 weeks | 0.04 | 0.06 | 0.11 |
| Expanded in blood after 6 weeks | 0.04 | 0.06 | 0.12 |
| Expanded in blood after both 2 and 6 weeks | 0.02 | 0.04 | 0.07 |

IQR; interquartile range

| **Supplementary Table S8** Percentage of unique and shared clonotypes between the largest 200 clonotypes at two time points: 2 weeks and 6 weeks after the CryoIT. The total clonal space for this comparison is 400 clonotypes: the top 200 clonotypes in week 2 and the top 200 clonotypes in week 6. | | | | | |
| --- | --- | --- | --- | --- | --- |
| **Clonotypes** | **Min** | **IQR1** | **Median** | **IQR3** | **Max** |
| % shared between the two time points | 1.25 | 17.55 | 27.91 | 41.48 | 54.83 |
| % unique identified 2 weeks after treatment | 22.78 | 29.09 | 35.73 | 40.94 | 49.13 |
| % unique identified 6 weeks after treatment | 22.39 | 29.40 | 35.96 | 41.51 | 49.63 |

IQR; interquartile range

**Supplementary Figure S9** Venn diagrams depicting clonotypic overlap.

Each patient is represented by one four-way Venn diagram. The two circles to the left depict the clonotypes detected as new (blue) or expanded (yellow) two weeks after the CryoIT, while the number of clonotypes that were new (green) and expanded (red) after six weeks is found to the right. The counts of clonotypes found in two or more of the groups (range 0-5) are numbered in the intersections.


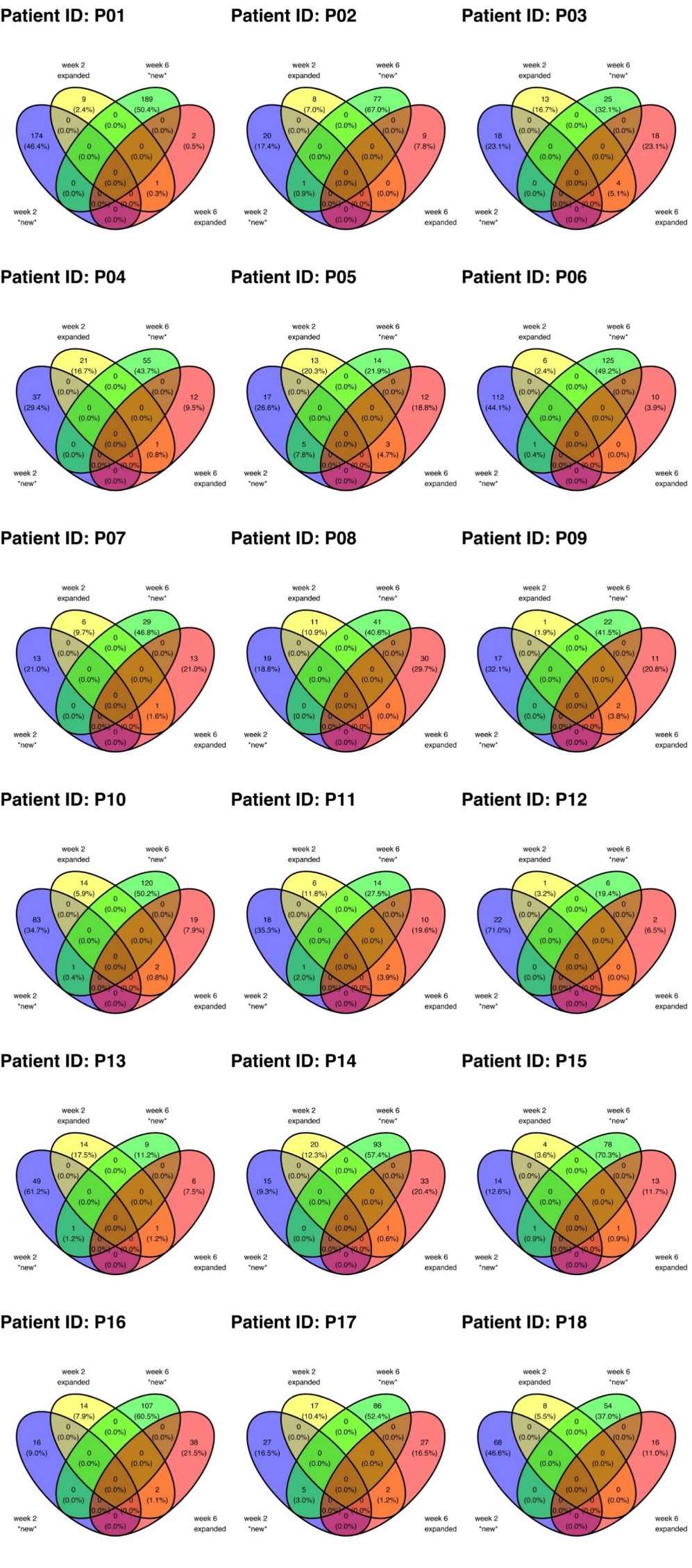


**Supplementary S12** Changes in immunological and routine laboratory variables

Peripheral blood samples, sequentially collected from the time of inclusion were examined in order to compare immunological and routine laboratory variables. From the nadir established during the last year prior to trial inclusion to the last measured PSA (at inclusion), 12/18 patients demonstrated PSA increases >100%, while four showed PSA increases >78%, and the last two had PSA-increases of 40-50% of the nadir values. The median number of days from nadir to trial inclusion were 233 (IQR 175-292).

**Supplementary Figure S10** Percent changes in the PSA levels from nadir.

Changes in PSA from the lowest levels measured (nadir) prior to participation to the time of inclusion are illustrated along the x-axis for all participants (n=18). Log10-transformed percentage change in PSA along the y-axis.


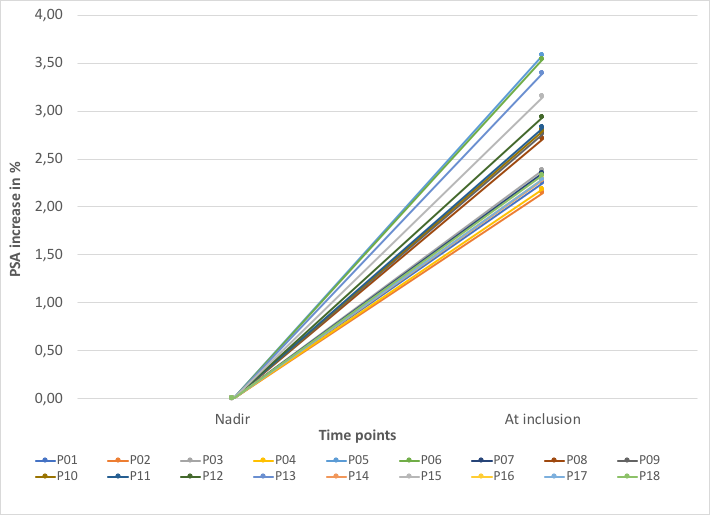


**Supplementary Table S9** Differences between response groups in median intra-patient blood value changes (%) after the CryoIT. Responses were estimated 22 weeks after the treatment.

|  | **Percentage change after 2 weeks** | | | **Percentage change after 6 weeks** | | |
| --- | --- | --- | --- | --- | --- | --- |
|  | **Patients with clinical benefit (n=6)** | **Non-responders (n=11)** | **P value** | **Patients with clinical benefit (n=6)** | **Non-responders (n=11)** | **P value** |
| Prostate specific antigen | -39.7 (-55.4;-29.9) | 55.0 (17.9;140.0) | 0.003 | -47.5 (-64.8;-46.1) | 52.8 (15.5;218.6) | 0.0019 |
| Alkaline phosphatase | -4.5 (-8.7;-0.5) | 14.5 (-3.6;49.8) | 0.11 | -7.3 (-11.5;-3.0) | 28.6 (-0.7;73.2) | 0.01 |
| Lactate dehydrogenase | 1.3 (-2.5;13.6) | 11.3 (-0.5;15.5) | 0.44 | 0.9 (-1.6;3.9) | 3.7 (-10.4;26.4) | 0.95 |
| C-reactive protein | 0.0(-33.3;100.0) | 100.0 (0.0;275.0) | 0.35 | 0.0 (-33.3;33.3) | 150.00 (1.0;462.5) | 0.07 |
| Platelets | 24.6 (21.5;29.8) | 28.1 (24.0;37.5) | 0.46 | -2.3 (-4.8;1.8) | 8.0 (-6.1;17.8) | 0.40 |
| Hemoglobin | -4.4 (-7.95;1.16) | -4.4 (-12.5;-2.0) | 0.43 | -4.4 (-5.7;-0.9) | -7.2 (-11.6;-4.9) | 0.19 |
| Leukocytes | -0.22 (-22.46;12.21) | 2.42 (-3.26;8.45) | 1.0 | -28.89 (-32.01;-11.39) | -16.58 (-26.36;-15.80) | 0.49 |
| Neutrophils | 0.38 (-26.1;17.2) | 5.56 (0.98;21.22) | 0·69 | -27.72 (-34.52;11.16) | -19.08 (-23.50;-15.20) | 0.56 |
| Lymphocytes | -9.38 (-25.35;-1.56) | -16.67 (-24.27;-6.51) | 0.76 | -28.52 (-38.11;-19.06) | -21.43 (-36.40;-14.06) | 0.66 |
| Monocytes | -1.7 (-10.3;10.8) | 0.0 (-3.5;6.0) | 0.96 | -4.4 (-28.3;5.1) | 7.6 (-14.1;14.5) | 0.40 |
| Basophils | NA | NA | NA | NA | NA | NA |
| Eosinophils | 50.0 (0.0;100.0) | NA | NA | 0.0 (-37.5;75.0) | NA | NA |
| Sodium | 0.00 (-0.53;0.00) | -1.06 (-1.92;0.00) | 0.15 | 0.32 (-0.53;0.71) | -1.43 (-2.11;0.00) | 0.08 |
| Potassium | 1.52 (-1.67;4.33) | 2.38 (0.00;7.90) | 0.68 | 1.11 (-1.71;7.70) | 2.22 (-3.39;4.97) | 0.92 |
| Calcium | -0.36 (-1.59;0.00) | -0.41 (-2.65;1.45) | 0.83 | -2.99 (-3.57;-0.85) | -0.41 (-3.35;1.68) | 0.27 |
| Albumin | 0.00 (-6.82;1.50) | 0.00 (-2.22;0.00) | 1.0 | -3.22 (-7.04;-0.57) | -6.19 (-8.12;-1.14) | 0.91 |
| Alanine aminotransferase | -13.38 (-26.56;11.48) | -9.52 (-27.71;4.17) | 1.0 | -11.15 (-22.34;-1.09) | -13.89 (-30.38;4.83) | 0.88 |
| Gamma-glutamyl transferase | -9.52 (-14.82;6.90) | 3.45 (-15.90;34.74) | 0.51 | -17.24 (-21.95;0.00) | 21.74 (-6.35;47.20) | 0.09 |
| Ratio CD4 positive / CD8 positive lymphocytes | 1.68 (-5.05;1.37) | -3.79 (-18.61;4.00) | 0.59 | -5.22 (-12.14;-4.38) | -20.30 (-29.16;0.96) | 0.30 |
| CD3 positive leukocytes (%) | 5.65 (0.57;7.74) | 1.30 (-1.10;9.69) | 0.81 | 4.70 (2.67;7.91) | 4.07 (2.24;13.84) | 0.73 |
| CD3 positive leukocytes (n) | -14.72 (-23.60;-2.81) | -21.89 (-29.60;-14.12) | 0.40 | -31.17 (-39.23;-11.92) | -25.55 (-26.52;3.16) | 0.88 |
| CD4 positive lymphocytes (%) | 4.31 (0.27;10.64) | 3.08 (-7.01;8.27) | 0·46 | 2.34 (-2.86;8.84) | -1.13 (-5.06;10.54) | 0.66 |
| CD4 positive lymphocytes (n) | -24.82 (-28.83;13.86) | -32.55 (-34.67;-16.33) | 0.40 | -32.55 (-34.67;-16.33) | -31.84 (-35.67;4.79) | 0.88 |
| CD8 positive lymphocytes (%) | 5.52 (-1.51;11.40) | 9.09 (-0.64;20.56) | 0.59 | 13.07 (5.76;14.71) | 18.72 (14.56;36.60) | 0.18 |
| CD8 positive lymphocytes (n) | -13.40 (-28.24;1.14) | -20.00 (-27.14;-4.21) | 0.81 | -25.19 (-37.34;-6.01) | -11.72 (-33.38;5.03) | 0.59 |
| Natural killer cells (%) | -7.46 (-22.58;28.49) | -19.59 (-25.11;33.46) | 0.96 | -2.14 (-6.62;15.42) | -11.11 (-27.80;34.50) | 0.73 |
| Natural killer cells (n) | -19.99 (-42.62;-4.69) | -21.62 (-39.34;-6.20) | 0.96 | -32.23 (-39.58;-11.96) | -35.48 (-45.51;-10.64) | 0.73 |
| Regulatory T lymphocytes (%) | 55.04 (39.49;126.79) | 30.42 (-40.56;6.16.67) | 0.87 | 25.00 (-5.88;60.98) | -4.00 (-28.32;1100.00) | 0.08 |

**References**

1. National Cancer Institute, Cancer Therapy Evaluation Program. Common toxicity criteria manual, Common Toxicity Criteria, Version 2.0, June 1, 1999.
2. Subklewe M, Geiger C, Lichtenegger FS, et al. New generation dendritic cell vaccine for immunotherapy of acute myeloid leukemia. Cancer Immunol Immunother. 2014; 63(10):1093-103. doi: 10.1007/s00262-014-1600-5
3. Zobywalski A, Javorovic M, Frankenberger B, et al. Generation of clinical grade dendritic cells with capacity to produce biologically active IL-12p70. J Transl Med. 2007; 5:18. doi: 10.1186/1479-5876-5-18
4. Jarnjak-Jankovic S, Hammerstad H, Saebøe-Larssen S, et al. A full scale comparative study of methods for generation of functional Dendritic cells for use as cancer vaccines. BMC Cancer. 2007; 7:119. doi: 10.1186/1471-2407-7-119
5. Ribas A. Tumor immunotherapy directed at PD-1. N Engl J Med. 2012 Jun 28;366(26):2517-9. doi: 10.1056/NEJMe1205943
6. McDermott J, Jimeno A. Pembrolizumab: PD-1 inhibition as a therapeutic strategy in cancer. Drugs Today (Barc). 2015 Jan;51(1):7-20. doi: 10.1358/dot.2015.51.1.2250387
7. Aaronson NK, Ahmedzai S, Bergman B, et al. The European Organization for Research and Treatment of Cancer QLQ-C30: a quality-of-life instrument for use in international clinical trials in oncology [Multicenter Study]. J Natl Cancer Inst. 1993; 85(5):365-76
8. Fayers P, Aaronson N, Bjordal K, et al. The EORTC QLQ-C30 Scoring Manual (3rd edition). Brussels: European Organisation for Research and Treatment of Cancer; 2001
9. Aarstad HJ, Aarstad AK, Birkhaug EJ, et al. The personality and quality of life in HNSCC patients following treatment. Eur J Cancer. 2003; 39(13):1852-60
10. Aarstad HJ, Aarstad AKH, Lybak S, et al. The amount of treatment versus quality of life in patients formerly treated for head and neck squamous cell carcinomas. European Archives of Oto-Rhino-Laryngology and Head & Neck. 2006; 2006/01/01;263(1):9-15. doi: 10.1007/s00405-005-0961-y
11. Aarstad AK, Aarstad HJ, Olofsson J. Personality and choice of coping predict quality of life in head and neck cancer patients during follow-up. Acta Oncol. 2008; 47(5):879-90. doi: 10.1080/02841860701798858
12. Hinz A, Einenkel J, Briest S, et al. Is it useful to calculate sum scores of the quality of life questionnaire EORTC QLQ-C30? Eur J Cancer Care (Engl). 2012; 21(5):677-83. doi: 10.1111/j.1365-2354.2012.01367.x

Seitz V, Schaper S, Dröge A, Lenze D, Hummel M, Hennig S. A new method to prevent carry-over contaminations in two-step PCR NGS library preparations. Nucleic Acids Res. 2015 Nov 16;43(20):e135. doi: 10.1093/nar/gkv694

R Core Team, R: A Language and Environment for Statistical Computing. 2019.
